# Optimizing Cancer Vaccinations Using a Physiologically‐Based Pharmacokinetic/Pharmacodynamic (PBPK/PD) Model

**DOI:** 10.1002/advs.78044

**Published:** 2026-09-30

**Authors:** Mohammad R. Nikmaneshi, Timothy P. Padera, Lance L. Munn

**Affiliations:** ^1^ Department of Radiation Oncology Massachusetts General Hospital and Harvard Medical School Boston Massachusetts USA

**Keywords:** antigen, cancer vaccination, overall immune hotness, suppressive factors, systemic immunity, tumor immune hotness

## Abstract

Antigen‐based tumor vaccines rely on adjuvants to stimulate local inflammation, recruit antigen‐presenting cells (APCs), and enhance immune activation. However, the complex interplay between antigen transport, lymphatic drainage, and immune cell dynamics across organs remains poorly understood, limiting the rational design of vaccination strategies. Here, we present a multiscale compartmental Physiologically Based Pharmacokinetic/Pharmacodynamic (PBPK/PD) model of antigen vaccination that integrates systemic circulation, lymphatic connectivity, and immune cell activation at the whole‐body level. The model incorporates arterial, venous, and lymphatic flows, organ‐specific interstitium and lymph node (LN) networks, and a superficial skin network. The model reproduces spatiotemporal distributions of antigen and suppressive factors, APC activation, and nT priming across activation sites, including LNs and spleen. Our results show that the sensitivity of vaccination‐induced immunity is highly related to antigen and suppressive factor production by the tumor, and that early‐stage vaccination enhances immunity. Since the model is able to identify optimal vaccination administration over the course of tumor growth for each patient with certain levels of antigen and immunosuppressive factors, it can serve as the foundation for digital twins of patients to help inform anti‐cancer vaccination strategies.

## Graphical abstract

Where and when should a cancer vaccine be administered? A whole‐body PBPK model demonstrates that systemic immune transport critically determines vaccination efficacy. By simulating how organ‐specific immune interactions influence treatment response, the study provides a quantitative framework for optimizing cancer vaccination strategies.

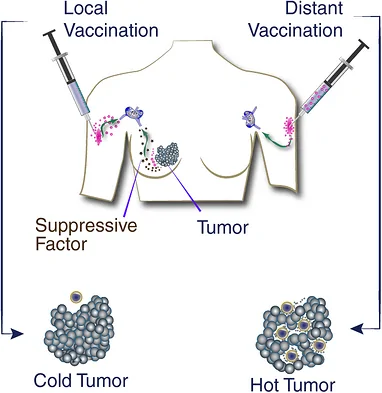

## 1 Introduction

Despite encouraging results from cancer vaccines—particularly mRNA‐based and antigen‐based platforms—patient responses remain highly variable, even among individuals with the same tumor type and stage [1, 2, 3, 4]. Preclinical and animal studies have consistently demonstrated considerable fluctuations in response to immunotherapy, even within genetically identical models [3, 5]. One of the main reasons for this inconsistency is the complex and heterogeneous trafficking of T cells to the tumor and their variable infiltration patterns, an aspect captured by the concept of tumor‐immune “hotness,” which refers to the quality and spatial distribution of T cell infiltration within the tumor microenvironment (TME). Tumors are generally classified immunologically into three categories: immune‐desert or “cold” tumors characterized by absent or non‐activated T cells; immune‐excluded or non‐inflamed “cold” tumors in which T cells are present but fail to infiltrate the tumor parenchyma; and immune‐inflamed or “hot” tumors that are enriched with infiltrating, activated T cells [6, 7, 8, 9, 10]. The effectiveness of cancer vaccination can therefore be strongly influenced by the tumor's hotness.

In addition to the tumor properties, three major factors —the vaccination site, timing of administration, and type of vaccination— may play critical roles in determining the efficacy of cancer vaccination. As a tumor grows, the distributions of immune agents such as antigen‐presenting cells (APCs), antigens, naïve T cells, and cytokines undergo dynamic changes throughout the body [11, 12]. Consequently, the potential for T cell activation, which depends on pro‐ and anti‐inflammatory signals, also varies with time and location in the body. Understanding the spatiotemporal distributions of these components is crucial to the success of any immunization strategy, including cancer vaccines.

Beyond local immune evasion at the tumor site, tumors can exert systemic immunosuppressive effects by releasing soluble factors that interfere with immune priming in secondary lymphoid organs. For example, tumor‐derived cytokines and metabolites such as TGF‐β, IL‐10, VEGF, and lactic acid have been shown to suppress dendritic cell (DC) maturation and function, impairing antigen presentation and co‐stimulation required for naive T cell activation in lymph nodes [13, 14, 15, 16, 17]. These factors can downregulate MHC and costimulatory molecules (e.g., CD80, CD86) on APCs, inhibit their migration to lymph nodes, and promote the development of tolerogenic DCs. In parallel, they can directly inhibit T cell receptor signaling, reduce IL‐2 production, and promote anergy or regulatory phenotypes in naive T cells, even before they reach the tumor [12, 18, 19, 20]. This pre‐conditioning of the systemic immune environment compromises the initiation of effective anti‐tumor responses and may contribute to poor responsiveness to immunotherapies.

Common routes of vaccine administration, such as pulmonary delivery via inhalation, oral or gastrointestinal (GI) delivery, subcutaneous injection, and intramuscular injection, each engage the immune system differently depending on the tumor's location, stage, and systemic immunosuppressive activity [21, 22, 23, 24, 25]. Key physiological and anatomical variables—including blood and lymphatic circulation, tissue‐specific architecture, lymphatic connectivity, and tissue transport properties—strongly affect the distribution of immune components and thus the resulting vaccine performance [26, 27, 28, 29, 30]. These complexities mean that cancer vaccination outcomes can be highly variable and may differ significantly not only between species but also among individual patients. Due to the anatomical and physiological differences between humans and animals, preclinical animal models often fail to recapitulate human vaccination responses accurately [31, 32, 33, 34]. Furthermore, there is currently no clinically available tool to optimize vaccination strategies for individual cancer patients.

In this context, computational modeling emerges as a powerful and efficient approach to predict patient‐specific immune responses to vaccination, with minimal time investment and high biological relevance. Although several mathematical models have been developed to simulate the systemic trafficking of immune cells and the local immune dynamics in the tumor microenvironment [35, 36, 37, 38, 39, 40], a comprehensive whole‐body model that incorporates both systemic immunity and cancer metabolism remains unavailable. Such a model is critical for generating a holistic understanding of the immune landscape and for predicting how the body will respond to various vaccination strategies—including variations in timing, site, and vaccine type—while accounting for the tumor's biological behavior and stage.

The study of systemic immune responses to immunization requires comprehensive models that capture the complexity of immune dynamics throughout the entire body. Recently, Physiologically‐based pharmacokinetic/pharmacodynamic (PBPK/PD) models and multi‐compartmental frameworks have been developed to map the immune system's behavior across organs, incorporating vascular and lymphatic connectivity [37, 41, 42, 43]. These models are instrumental in addressing critical questions about the timing, magnitude, and localization of immune responses to various cancer therapies, including vaccination, checkpoint blockade, adoptive T cell therapy such as CAR‐T, and radiotherapy [37, 44]. Recent advances in PBPK modeling have expanded its applications in oncology drug development, pharmacokinetic prediction, and translational cancer research. PBPK frameworks have been used to evaluate pharmacokinetics in cancer patients [45], improve oncology drug development pipelines [46], and perform cross‐species risk assessment [47]. Hybrid PBPK approaches integrating mechanistic and data‐driven modeling have also recently emerged [48]. However, most existing PBPK studies primarily focus on drug pharmacokinetics, while fewer models integrate systemic immune‐cell trafficking, lymphatic/LN activation, tumor‐induced immunosuppression, and tumor–immune interactions within a unified whole‐body framework.

Some early models simplify systemic immunity by including only a few compartments—typically the target organ (e.g., tumor), the bloodstream, and a single lymph node—serving as the site for T cell priming [49, 50, 51, 52]. While useful for initial insights, these models often fail to capture the heterogeneity of immune cell trafficking, recirculation, and clearance mechanisms that can delay or dampen immune activation in patients. More advanced approaches incorporate multiple lymphoid and non‐lymphoid compartments to better resolve immune cell kinetics, account for physiological clearance, and improve prediction of treatment outcomes [37, 53, 54]. Recent innovations in systems immunology have aimed to bridge these gaps by developing integrated models that simulate the spatiotemporal dynamics of immune responses at systemic levels. These include models that account for organ‐specific lymphatic drainage, allowing simultaneous tracking of T cell activation across multiple lymph nodes and their migration toward tumors [37, 55]. Such frameworks are particularly valuable for studying responses to combination therapies, like radiotherapy (RT) and vaccination, which can initiate immune activation in distinct anatomical contexts [56, 57]. Additionally, the heterogeneity of organ‐specific environments—ranging from mechanical properties and vascular permeability to cytokine gradients and antigen presentation efficiency—adds further complexity to accurately predicting immune responses [58, 59, 60, 61, 62, 63, 64]. Simplified models often overlook these factors, leading to poor translational relevance in predicting post‐vaccination immunity or T cell‐based therapy outcomes. Therefore, incorporating organ‐specific immunobiology, lymphatic topology, and dynamic intercompartmental feedback is essential for building predictive, clinically relevant models of immunotherapy. Another issue with current PBPK models is their lack of species specificity. It is often unclear whether the model has been developed for humans, mice, or another species [47]. Since each species has distinct anatomical and physiological characteristics, using a single model for both humans and mice can lead to irrelevant results and poor translational outcomes for patients.

In general, existing PBPK/PD models of immunity have several key limitations: (1) they lack organ‐specific lymph node (LN) activation with a dynamic range, limiting their ability to capture systemic heterogeneity in immune cell activation—an essential feature for models aimed at optimizing vaccination, immune checkpoint blockade (ICB), and cell therapies such as CAR‐T; (2) they often rely on an oversimplified representation of the superficial network as a single “skin” compartment, which neglects the cross‐talk between organ‐specific LNs and superficial LNs and prevents modeling of site‐specific vaccination or infusion within the skin—an important consideration for cancers such as melanoma, where spatial variations can influence local pressure and, consequently, vaccination response; (3) they do not consistently represent each organ with its corresponding LNs, limiting the model's ability to distinguish species‐specific or organ‐specific immune dynamics; and (4) they lack comprehensive vaccination modeling in a systemic, human‐based framework that incorporates both transport and signaling barriers governing the activation of multiple immune cell populations, including antigen‐presenting cells (APCs) and T cells.

To address these gaps, we have developed a computational systems immunology model of human immuno‐physiology using a compartmental PBPK/PD approach that integrates the circulatory and lymphatic systems, major internal organs, the superficial immune network of the skin, lymph node architecture, immune cell trafficking, and tumor metabolism (Figure 1). The model uses a matrix‐based spatial framework that allows dynamic configuration of interconnected compartments in the human body. Each compartment includes vascular and tissue‐level structures responsible for interstitial fluid flow and molecular transport and is linked to lymph nodes either in parallel or in series, capturing physiologically accurate transport pathways. The skin is modeled as a special organ and includes multiple interconnected subcompartments, each with draining lymph nodes that are also integrated into the systemic network, thereby linking peripheral immune responses to systemic immunity. This whole‐body framework serves as a virtual platform for simulating and optimizing cancer vaccination strategies in a patient‐specific manner across spatial and temporal scales.

**FIGURE 1:**
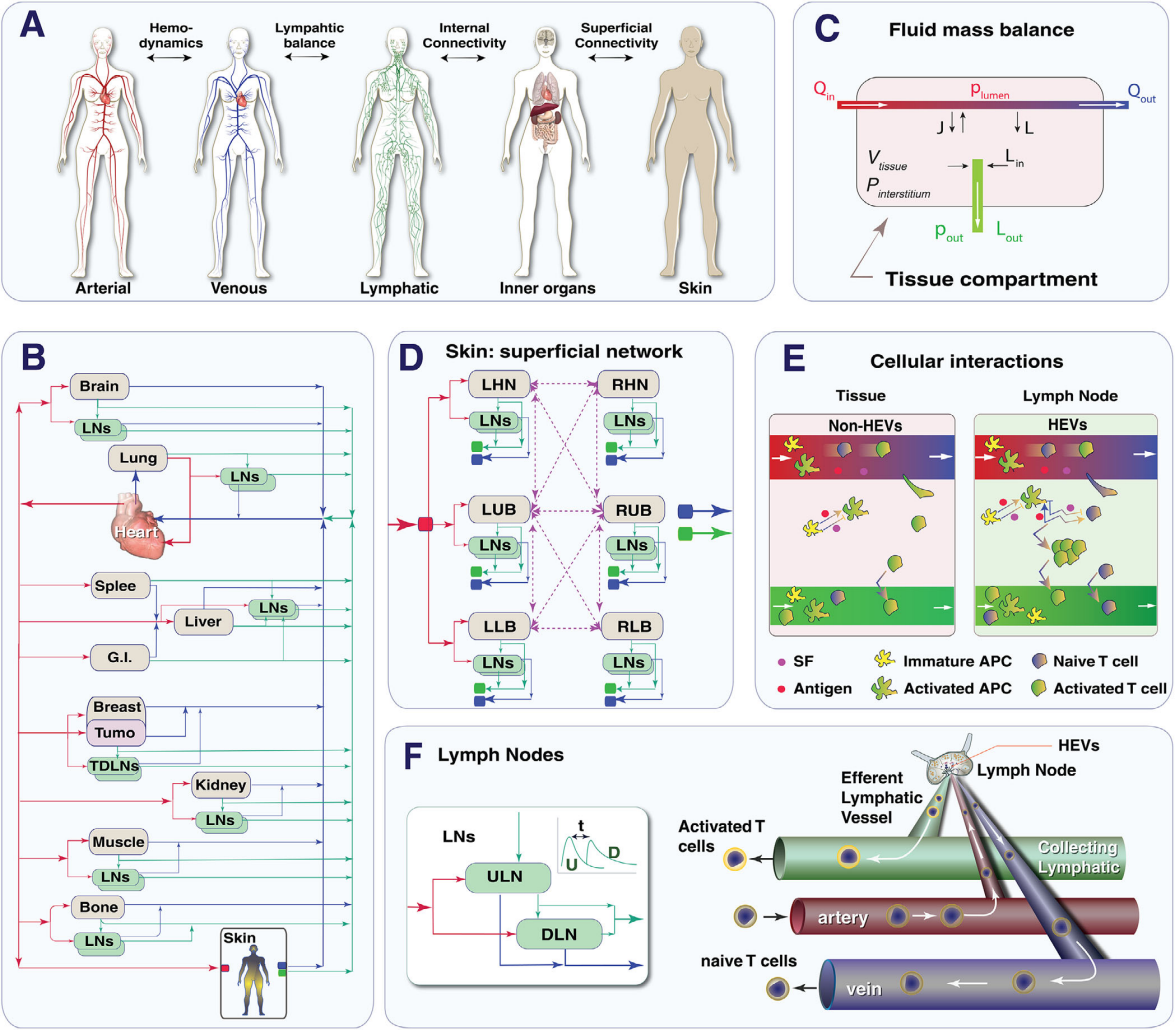
Model configuration and computational PBPK/PD model. (A) The model integrates multiple body layers, including the arterial, venous, and lymphatic systems, internal organs, and skin. (B) The computational diagram illustrates how individual internal organs, represented as separate compartments, are connected via arterial (red arrows) and venous (blue arrows) circulations, as well as lymphatic drainage (green arrows). (C) Fluid mass balance scheme. Tissues are modeled as separate compartments, and each compartment has its own fluid mass balance through blood vessels, interstitium, and the lymphatic system. (D). Skin has its own superficial network, including different lymphosomes interconnected with superficial flows (dashed‐purple arrows) and also interconnected with other inner organs through blood and lymphatic local recirculation. (E). Naive T cells can be activated by APCs in LNs but not in other tissues. (F). Each compartment contains its own LN network, consisting of upstream lymph nodes (ULNs) and downstream lymph nodes (DLNs). Each LN includes all physiological pathways: afferent and efferent lymphatic vessels, and blood circulation through high endothelial venules (HEVs).

## 2 Methods

### 2.1 Scales and Agents of the Model

The components of the compartmental PBPK/PD model are illustrated in Figure 1. The model is multiscale, including body‐scale, cellular‐scale, and molecular‐scale.

At the *body scale*, the model incorporates four layers: arterial, venous, and lymphatic circulations, and organs, which include the internal organs and superficial (skin) tissue (Figure 1A). The major internal organs include the heart (with distinct left and right ventricles), brain, lungs, liver, spleen, GI tract, kidneys, breast tissue, skeletal muscle, and bone. The superficial skin network is divided into six regions: left and right head‐neck (LHN and RHN), left and right upper body (LUB and RUB), and left and right lower body (LLB and RLB). The tumor compartment can be embedded in any organ, including the skin. The computational chart shows inner organs and skin compartments with their associated draining LNs interconnected by arterial (red arrows), venous (blue arrows), lymphatic (green arrows), and superficial interstitial (purple dashed arrows) flows (Figure 1B). Each compartment includes tissue, blood vessels, and lymphatic vessels (Figure 1C).

Using hemodynamic parameters of each compartment—including blood flow rate (Q), luminal pressure, vessel wall permeability, and porosity—the arterial and venous flows are coupled with lymphatic flows throughout the system. A modified Starling's law with interstitial fluid pressure and oncotic pressure determined by vessel wall permeability/porosity defines the lymphatic balance (Figure 1C). The coupled blood–lymphatic system provides internal connectivity among inner organs (Figure 1B) and superficial connectivity across skin compartments, which in turn link to inner organs (Figure 1D). Each organ receives physiological blood flow based on known flow rates, and organ‐specific transport properties regulate exchanges of cells and molecules between the vasculature and interstitium. This framework allows the model to simulate dynamic infiltration and recirculation of immune components between systemic compartments and target tissues, providing a platform to explore vaccination strategies and immune responses across the whole body. Additional details are provided in the Supporting Information.

At the *cellular scale*, the model includes tumor cells and angiogenic vasculature, naive T cells (nTs), effector T cells (eTs), immature antigen‐presenting cells (imAPCs), and activated antigen‐presenting cells (aAPCs) (Figure 1E). CD4/CD8 post‐activation ratios are assumed constant among eTs (2:1). nTs are generated in the thymus, modeled as a variable source with cytokines directly injected into the left ventricle. Generation of imAPCs mainly occurs in bone marrow and, to a lesser extent, in skin, and is affected by cytokine levels.

At the *molecular scale*, the model simulates transport and interactions of antigens, cytokines, VEGF, IL‐2, and a putative tumor‐derived suppressive factor (SF). Due to limited quantitative data on the relative contributions of individual suppressive pathways, tumor‐induced suppression is modeled as an aggregate factor. While this enables a tractable system‐level analysis, it does not capture pathway‐specific effects. Antigen is released by cancer cells or supplied via vaccination; inflammatory cytokines are considered as a single species, which is secreted strongly by aAPCs and moderately by imAPCs and other immune cells (assumed a constant source for other immune cells here). VEGF is released by tumor cells to promote angiogenesis; IL‐2 is modeled separately and is secreted by activated effector T cells (eTs), including both CD4 and CD8 subsets, with CD4 eTs serving as the primary source; SF is released by cancer cells.

### 2.2 Vaccine Model

The vaccine is modeled as an antigen source administered to the target tissue using a Dirac delta function. Because therapeutic cancer vaccines are typically formulated with adjuvants to enhance innate immune activation, adjuvant effects are incorporated implicitly rather than modeled as a separate species. Accordingly, the vaccine is represented as an additional source of antigen, and APC activation by vaccine‐derived antigen is assumed to capture the combined effects of antigen recognition and adjuvant‐mediated immune stimulation. Tumor‐derived and vaccine‐derived antigens are represented by the same antigen species and therefore activate APCs through the same model equations. Consequently, vaccination increases local antigen availability, thereby enhancing APC activation, cytokine production, and subsequent T‐cell priming.

### 2.3 Inhibition by Tumor‐Produced Suppressive Factors

Since cancer can release many types of metabolites, including suppressive factors (SFs) that inhibit T cell activation, understanding their role in modulating immune response is essential. Although the precise mechanisms by which SFs influence T cell priming remain unclear, we assume in this model that they affect both the activation of antigen‐presenting cells (APCs) and the ability of the activated APCs to activate naïve T cells (nTs). Therefore, in addition to tracking the distributions of antigens, nTs, and APCs within lymph nodes (LNs), the distribution of SFs within the LN network is considered a critical parameter in determining the extent of systemic T cell activation.

### 2.4 Cellular Dynamics

#### 2.4.1 Combining Cellular and Molecular Scales

imAPCs and aAPCs, nTs, and eTs interact differently within the tissue interstitium compared to the LN interstitium (Figure 1E). APCs can traffic to both tissue and LN interstitium. nTs can enter the LN interstitium but not the tissue interstitium [65, 66, 67]. eTs can be generated and proliferate in the LN interstitium when nTs interact with aAPCs. Activation of nTs occurs when antigen level is sufficient, and SF level is low; eTs can exit the blood and migrate within tissue compartments, but do not enter LNs. eT cells can enter the tumor tissue and interact with cancer cells. As we are only interested in the initial activation process and the accumulation of T cells in the tumor, we neglect immunoregulatory mechanisms of T cells once they enter the tumor tissue, such as exhaustion. We also neglect later phases of immune response, such as memory cell formation and circulation. Key immune interactions include APC activation mostly in peripheral tissues, which depends on local antigen, cytokine, and suppressive factor (SF) levels, followed by the activation of naïve T cells (nTs) in secondary lymphoid organs, including the spleen and lymph nodes (LNs) (Figure 1E). ImAPCs circulate through the blood vasculature and home to the peripheral compartments with elevated antigen levels, where they can become activated. aAPCs subsequently migrate through the lymphatic circulation to LNs and spleen, where they co‐localize with nTs and initiate their activation. Both APC activation and nT priming can be inhibited by SF.

### 2.5 T Cells Activation Sites

In general, LNs and spleen are activation sites for nTs. Each organ has multiple draining LNs with the potential for different antigen, cytokine and SF levels in each. We simplify the system by including two lymph nodes arranged in series that drain each organ, notated as the upstream LNs (ULNs) and downstream LNs (DLNs) (Figure 1F). Each LN has its own blood circulation, and cell trafficking through High endothelial venules (HEVs) as well as afferent/efferent pathways of lymphatic circulation, are considered independently (Figure 1F).

### 2.6 Governing Equations of the Multiscale PBPK/PD Framework

Based on the physiological and cellular framework described above, we developed a system of coupled compartmental pharmacokinetic‐pharmacodynamic (PBPK/PD) equations to describe trafficking, activation, proliferation, suppression, and tumor–immune interactions across organs, tissues, and lymph nodes (LNs). The model integrates body‐scale transport, cellular‐scale immune dynamics, and molecular‐scale signaling within physiologically connected compartments. Full organ‐specific equations and parameter definitions are provided in the Supporting Information.

### 2.7 Body‐Scale Mass Transport

At the body scale, the model describes systemic transport of cells and molecules through arterial, venous, lymphatic, tissue, and LN compartments. For each agent *c_i_*, mass conservation governs blood perfusion, endothelial attachment/detachment, transmigration, and lymphatic drainage between organs and lymphoid tissues.

The generalized vascular transport equation for cellular agents is:

$$\begin{array}{ccc} \frac{dc_{i}^{b}}{dt} & = & \mathrm{blood}\;\mathrm{inflow}-\mathrm{blood}\;\mathrm{outflow}-\mathrm{endothelial}\;\mathrm{attachment}\\ & & +\;\mathrm{detachment} \end{array}$$

While the mass balance for the cells captured on the vessel wall is:

$$\begin{array}{ccc} \frac{dc_{i}^{c}}{dt} & = & \mathrm{endothelial}\;\mathrm{attachment}+\mathrm{detachment}\;\\ & & -\;\;\mathrm{Transvascular}\;\mathrm{transport} \end{array}$$

Molecular agents do not undergo a captured state; therefore, their transvascular transport is described by the following generalized transport equation:

$$\;\frac{dc_{i}^{b}}{dt}=\mathrm{blood}\;\mathrm{inflow}-\mathrm{blood}\;\mathrm{outflow}-\mathrm{Transvascular}\;\mathrm{transport}$$

While generalized interstitial transport for both molecular and cellular agents is governed by:

$$\begin{array}{ccc} \frac{dc_{i}^{ins}}{dt} & = & \mathrm{Transvascular}\;\mathrm{transport}\;-\mathrm{lymphatic}\;\mathrm{drainage}\\ & & +\;\mathrm{local}\;\mathrm{reactions} \end{array}$$

Transvascular transport of cellular agents is modeled exclusively by transmigration, whereas molecular agents are transported across the vessel wall by both convection and diffusion, described using a modified Starling‐based transport formulation that incorporates vascular permeability, interstitial pressure, oncotic pressure, and particle size. Organ‐specific blood and lymphatic flow connections determine the recirculation of immune cells and molecular species between tissues and draining LNs.

### 2.8 Cellular‐Scale Dynamics

At the cellular scale, the model describes activation, trafficking, proliferation, and tumor interactions of immune cells, including immature APCs (imAPCs), activated APCs (aAPCs), naïve T cells (nTs), and effector T cells (eTs). imAPCs are generated primarily in bone marrow and skin compartments and circulate systemically to tissue sites with elevated antigen levels. Upon antigen uptake, imAPCs can differentiate into aAPCs depending on local inflammatory cytokine and suppressive factor (SF) levels. Activated APCs subsequently migrate through lymphatic pathways into LNs, where they interact with nTs and promote their activation into eTs.

The generalized imAPC dynamics are:

$$\frac{dc_{imAPC}}{dt}=\mathrm{transport}+\mathrm{generation}-\mathrm{imAPC}\;\mathrm{activation}-\mathrm{decay}$$

Activated APC dynamics are governed by:

$$\frac{dc_{aAPC}}{dt}=\mathrm{transport}+\mathrm{imAPC}\;\mathrm{activation}-\mathrm{decay}$$

Naïve T cells are generated in the thymus and circulate through blood and lymphoid compartments. Within LNs and spleen, nTs interact with aAPCs and differentiate into eTs under sufficient antigen stimulation and low SF levels:

$$\frac{dc_{nT}}{dt}=\mathrm{transport}+\mathrm{thymic}\;\mathrm{generation}-nT\;\mathrm{activation}-\mathrm{decay}$$

Effector T cells proliferate in response to IL‐2 signaling and subsequently infiltrate tumor tissue through vascular attachment and transmigration mechanisms:

$$\begin{array}{ccc} \frac{dc_{\mathrm{eT}}}{\mathrm{dt}} & = & \mathrm{transport}+\mathrm{nT}\;\mathrm{activation}+\mathrm{proliferation}\\ & & -\;\mathrm{tumor}\;\mathrm{binding}+\mathrm{tumor}\;\mathrm{un}\mathrm{binding}-\mathrm{decay} \end{array}$$

Tumor growth depends on both avascular and vascularized proliferation and is coupled to eT‐mediated killing through reversible tumor–T‐cell conjugate formation:

$$\begin{array}{ccc} \frac{\mathrm{dC}}{\mathrm{dt}} & = & \mathrm{tumor}\;\mathrm{proliferation}-\mathrm{eT}\;\mathrm{binding}+\mathrm{eT}\;\mathrm{un}\mathrm{binding}\\ & & -\;\mathrm{eT}\_\mathrm{mediated}\;\mathrm{killing} \end{array}$$

Tumor angiogenesis is regulated by VEGF‐dependent vascular growth and pruning mechanisms:

$$\frac{\mathrm{dv}}{\mathrm{dt}}=\mathrm{VEGF}\_\mathrm{induced}\;\mathrm{angiogenesis}-\mathrm{vascular}\;\mathrm{pruning}$$

### 2.9 Molecular‐Scale Dynamics

At the molecular scale, the model tracks systemic transport and local reactions of antigen, inflammatory cytokines, IL‐2, VEGF, and tumor‐derived suppressive factors (SFs). These molecules undergo secretion, uptake, transport, and decay across blood, tissue, and LN compartments.

The generalized molecular transport‐reaction equation is:

$$\frac{dc_{m}}{dt}=\mathrm{systemic}\;\mathrm{transport}+\mathrm{secretion}-\mathrm{uptake}-\mathrm{decay}$$

where secretion terms depend on the producing cell populations and uptake is modeled using saturable Michaelis–Menten‐type kinetics. SFs released by tumor cells suppress APC activation and T‐cell priming throughout the LN network, whereas IL‐2 produced by activated eTs promotes eT proliferation within lymphoid organs. Antigen released from tumor cells or introduced by vaccination stimulates APC activation and downstream adaptive immune responses.

## 3 Results and Discussion

In the simulations, we assumed that tumors exhibit varying levels of antigen and suppressive factor (SF) release rates. A baseline value of 10^−3^ min^−1^was assigned for both antigen and SF production. To evaluate the impact of parameter uncertainty and biological variability, these rates were varied over ranges of ±20% and ±40% relative to their baseline values. To demonstrate the various systemic mechanisms involved in systemic T cell activation, we first simulated vaccination in the left arm of a patient with a tumor in the right breast (Figure 2A). In this simulation, the vaccine was modeled as a Dirac delta input administered at day 9 of tumor growth, acting as a source of antigen. Antigen concentration was normalized such that its maximum value was set to 1, representing a typical effective dose rather than an absolute physical concentration.

**FIGURE 2:**
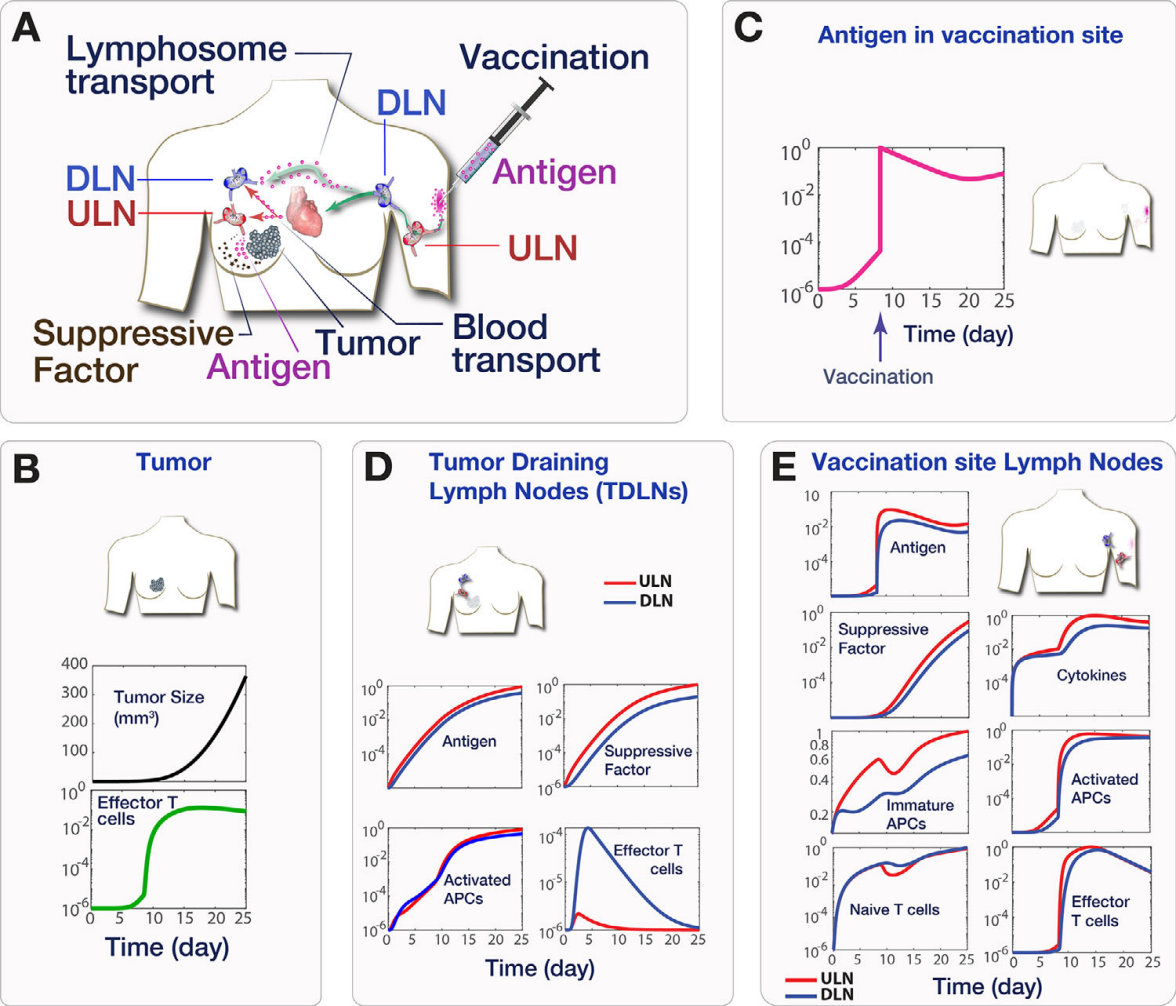
Mechanistic overview and temporal dynamics of tumor growth and immune response following a vaccination in the left upper body (LUB). (A) Schematic of the simulation. Vaccination is in the LUB; Antigen is produced by the tumor and is injected by the vaccination. In this simulation, the tumor is assumed to have the lowest antigen release rate (−40% of baseline = 0.6 × 10^−3^ min^−1^), and the vaccine serves as the main source of antigen, allowing us to clearly demonstrate the independent effect of the vaccine on immune activation; antigen and suppressive factor can transport through the lymphatic system as well as the bloodstream; we then monitor the dynamics in the various tissues over time. (B) Tumor growth and accumulation of effector T cells over the 25‐day period. Tumor growth reflects the increase in tumor tissue volume over time (Equation S9a). (C) Antigen level at the vaccination site. The sharp increase at day 9 is due to vaccination. (D) Antigen, effector T cell, activated APCs, and Suppressive Factor concentrations in the Upstream and downstream tumor‐draining lymph nodes (ULN, DLN, respectively). (E) Dynamics in the ULN an DLN that drain the vaccination site. Normalized levels of immature APCs, antigen, cytokines, suppressive factor, activated APCs, effector T cells and naive T cells over the 25‐day period. Antigen levels are normalized to the concentration in the vaccination site; SF levels are normalized to the concentration in the tumor tissue; cytokine levels are normalized to those in the vaccination site. APC levels are normalized to the baseline APC level in the bone. nT and eT levels are normalized to the initial nT population released from the thymus into the heart. The model assumes that tumor‐specific nTs are generated upon cancer initiation, and that the total nT population remains at steady‐state during the simulation. All y‐axes represent normalized concentrations and are displayed on a logarithmic scale.

Since cancer can release many types of metabolites, including suppressive factors (SFs) that inhibit T cell activation, understanding their role in modulating immune response is essential. Although the precise mechanisms by which SFs influence T cell priming remain unclear, we assume in this model that they affect both the activation of antigen‐presenting cells (APCs) and the ability of the activated APCs to activate naïve T cells (nTs). Therefore, in addition to tracking the distributions of antigens, nTs, and APCs within lymph nodes (LNs), the distribution of SFs within the LN network is considered a critical parameter in determining the extent of systemic T cell activation (Figure 2). Tumor growth is initiated on day 0 and progresses over 25 days (Figure 2B). As the tumor grows, its SFs and antigens are released into the systemic circulation (Figure 2A) reaching TDLNs and LNs. Antigen/adjuvant vaccination was administered subdermally one week after tumor initiation (i.e., when the tumor volume reached 15 mm^3^). It releases antigen into the local tissue, which subsequently drains into nearby LNs (both ULNs and DLNs). The temporal distribution of antigen in the vaccination site tissue was monitored until day 25 after tumor initiation (Figure 2C). In this simulation, the patient is assumed to have a low level of tumor‐derived antigen, which is negligible compared to the antigen introduced by the vaccine. Thus, immune activation is limited before vaccination. The results show the dynamics of key immune components in the ULNs and DLNs draining the tumor (Figure 2D) and the vaccination site (Figure 2E) for 16 days post‐vaccination, including antigen levels, SF levels, activated APCs (aAPCs), and effector T cells (eTs). Immature APCs (imAPCs) and naïve T cells (nTs) within the vaccination site draining LNs are also shown to monitor the dynamics of inactive immune cells (Figure 2E). Cytokines accumulation in the vaccination‐site LNs released by aAPCs, imAPCs, and other immune cells is also shown in Figure 2E. Finally, a fraction of the effector T cells activated in the vaccination‐site LNs, tumor‐draining LNs (TDLNs), and other LNs throughout the body infiltrate the tumor tissue (Figure 2B).

Immature APCs, produced mainly in the bone marrow and to a lesser extent in the skin, enter circulation and become activated primarily in peripheral tissues in response to local antigen and SF levels. APC activation may also occur in secondary lymphoid organs (spleen and LNs). Naïve T cells, released from the thymus into the blood circulation, are distributed systemically and accumulate in LNs, where they can differentiate into effector T cells following stimulation by aAPCs; this step is enhanced by the presence of antigen and inhibited by SF. Since this study focuses on T cell accumulation within the tumor microenvironment rather than cytotoxic function, activated T cells are assumed to be non‐cytotoxic. This assumption isolates trafficking and accumulation dynamics from downstream cytotoxic effects, which will be addressed in future extensions of the model.

Because the suppressive factor can modulate activation of APCs and T cells, it is important to consider its spatiotemporal variation after vaccination at various sites. While the tumor grows at any SF rate, the spatiotemporal distribution of SF in the LNs and spleen dynamically changes, thereby influencing the activation rates of APCs and T cells. To simplify the presentation of the results, we define a parameter – the activation potential (AP) of each tissue including LNs and spleen, which is the probability of activation occurring at that location. We assumed that AP is governed by an exponential function, with a maximum value of 1 when the SF concentration (*c_sf_*) is zero, and decaying toward 0 as *c_sf_* increases, with rate constant *k_sf_* (see the exponential term $e^{-k_{sf}c_{sf}}$ in Equations S15–S18). We can then see how different SF production rates affect the AP for the spleen, ULNs, and DLNs (Figure 3A). The AP is then integrated over the time course of the simulation (i.e., the area under the curve) to produce a total AP, (Figure 3B, polar plot). This analysis helps identify the appropriate time and site for vaccination at each stage of the tumor growth. For low SF, tumor LNs have some activation potential when the vaccination is administered early (until day 5 for ULNs and until day 7 for DLNs). However, increasing SF by 40% shortens the effective vaccination times to day 2 and 5, and then non‐activation and day 3 for ULNs and DLNs, respectively. Similar trends are seen for the spleen and LNs in other locations. In general, ULNs have lower activation potential, especially those draining the tumor, lung, G.I., muscle, and superficial skin LNs (LHN, RHN, LUB, RUB, LLB, RLB skins). Interestingly, in the LNs draining the lungs, the ULN has considerably less activation potential than the DLNs at every SF production rate. This is due to the fact that all blood (and SF) passes through the lungs, and the SF gets concentrated in the ULNs faster than the DLNs.

**FIGURE 3:**
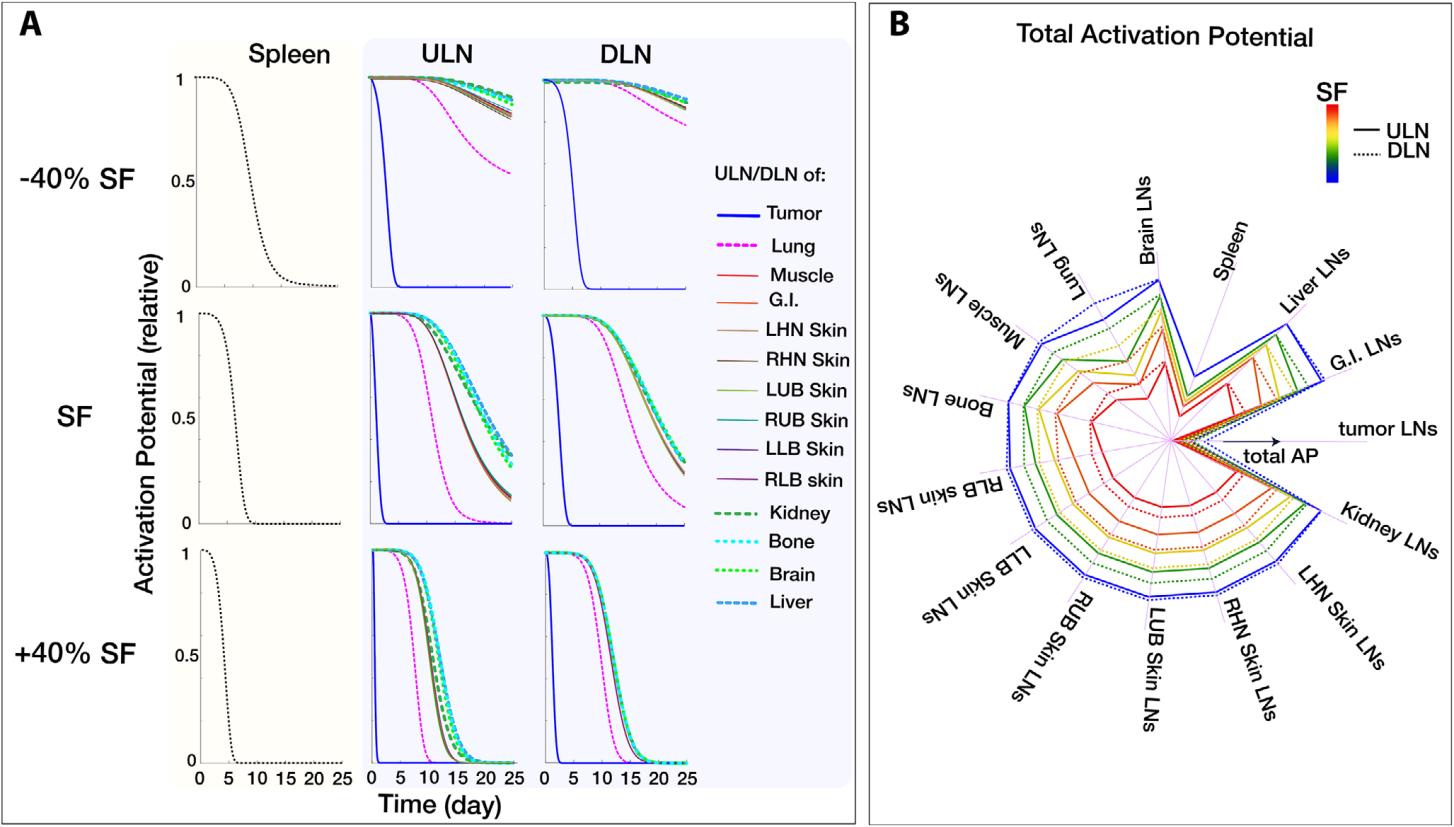
Activation potential (AP) of LNs and spleen. The baseline production rate of the suppressive factor (SF) by the tumor was set to 10^−3^ min^−1^ [68]. To evaluate the sensitivity of T‐cell activation to SFs, the SF production rate was varied by −40%, −20%, 0%, +20%, and +40% relative to the baseline value. (A) Temporal profiles of AP in the spleen and in the upstream (ULN) and downstream (DLN) lymph nodes of different organs for the minimum (−40%), baseline (0%), and maximum (+40%) SF production rates (10^−3^ min^−1^). (B) Area under the curve (AUC) of AP for all five SF production rates. Colors range from blue (minimum SF production rate) to red (maximum SF production rate), with yellow representing the baseline SF production rate. Solid and dashed lines correspond to ULNs and DLNs, respectively.

Post‐vaccination T cell activation is determined by the co‐localization of antigen, aAPCs, and naïve nTs within a LN, provided the LN is not suppressed by SF (Figure 3). This is illustrated in the scenario shown in Figure 1E: even when the tumor‐draining LNs have high antigen level and contain both nTs and aAPCs, an elevated SF concentration can prevent T cell activation (maximum normalized number of activated T cells in tumor‐draining DLN 10^−4^ logscale (Figure 1D) versus 10^0^ logscale for the vaccination LNs (Figure 1E)). As a result, eTs are instead generated in other, less suppressed LNs and subsequently must traffic through the vasculature and migrate into the tumor tissue (Figure 1).

To investigate how the vaccination site affects the systemic immune response, we introduced vaccine at various internal organs and skin regions and monitored the distribution of eT cells in all LNs and the spleen. We simulated responses in patients with breast tumors exhibiting varying levels of antigen and SF, including: (1) low antigen and low SF (−40% relative to the baseline antigen and SF production rates), (2) high antigen and low SF (+40% and −40% relative to the baseline antigen and SF production rates, respectively), (3) low antigen and high SF (−40% and +40% relative to the baseline antigen and SF production rates, respectively), and (4) high antigen and high SF (+40% relative to the baseline antigen and SF production rates).

To assess the efficiency of vaccination, we define three metrics: i) activation site occupancy (ASO), calculated as the area under the curve (AUC) of eT cell concentration for the spleen and each LN during the 2 weeks following vaccination (Figure 4), ii) Overall Immune Hotness (OIH), defined as the total number of eT cells integrated over all LNs and the spleen (Figure 5), and iii) Tumor Immune Hotness (TIH), defined as the fraction of all eT cells localized within the tumor (Figure 6). Thus, ASO quantifies activation at each immune activation site, OIH reflects the magnitude of systemic immune activation, and TIH represents the efficiency of effector T‐cell accumulation within the tumor and is therefore related to anti‐tumor cytotoxic potential.

**FIGURE 4:**
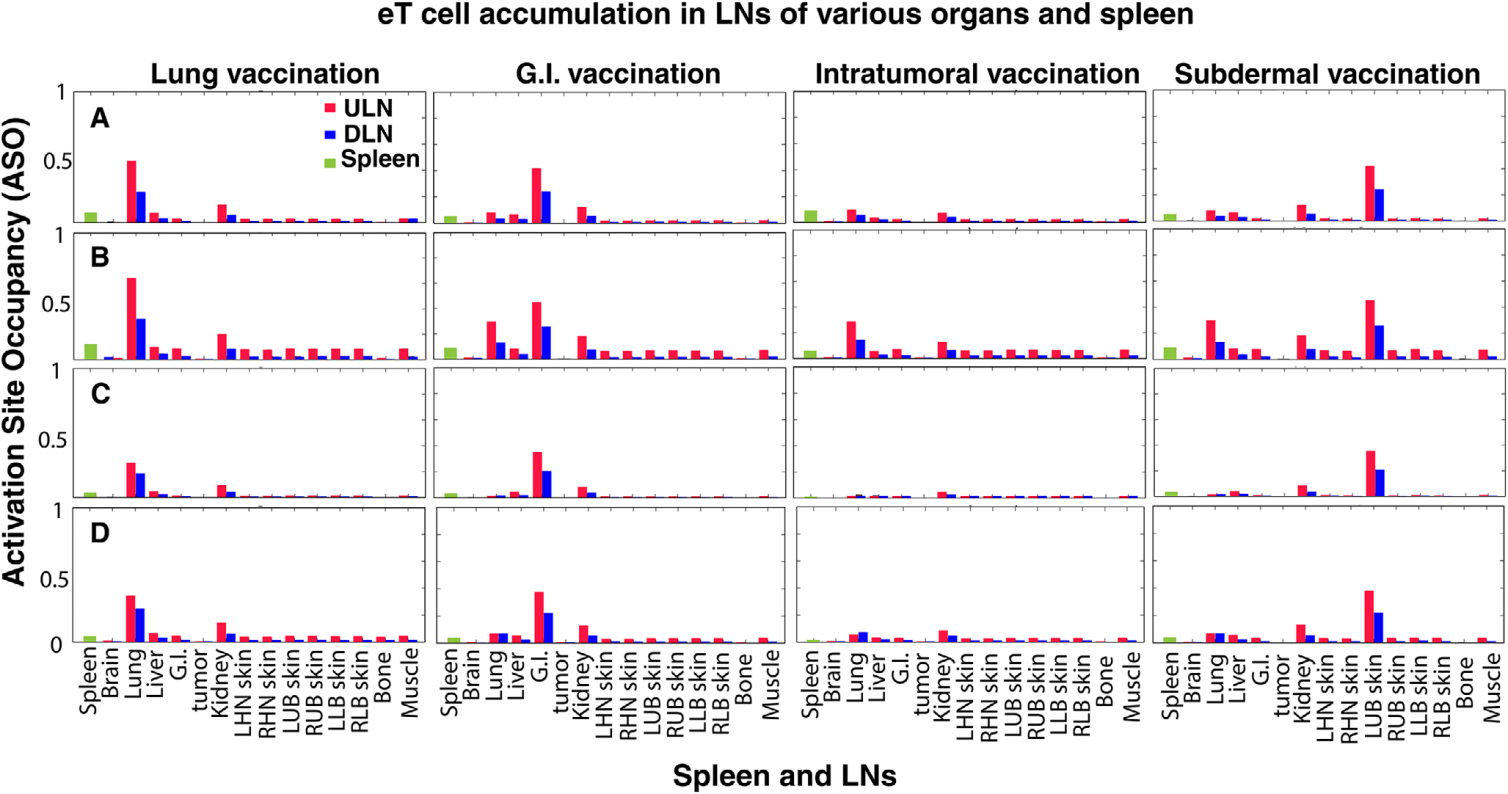
Accumulation of eT cells two weeks post‐immunization in the spleen and LNs of each organ, defined as activation site occupancy (ASO). From left to right, the vaccination sites are lung, gastrointestinal (G.I.), tumor, and left upper body (LUB) subdermal. (Row A) Patients with low tumor‐induced antigen and suppressive factor (SF) production rates, both set to −20% relative to baseline (i.e., 0.8 × 10^−3^ min^−1^). (Row B) Patients with a +20% increase in the tumor‐induced antigen production rate relative to Row A, while maintaining the same SF production rate. (Row C) Patients with the same antigen production rate as in Row A but a +20% increase in the SF production rate. (Row D) Patients with +20% increases in both tumor‐induced antigen and SF production rates relative to Row A. Note that eT‐cell accumulation in each lymph node (LN) represents the sum of eT cells in its upstream (ULN) and downstream (DLN) lymph nodes.

**FIGURE 5:**
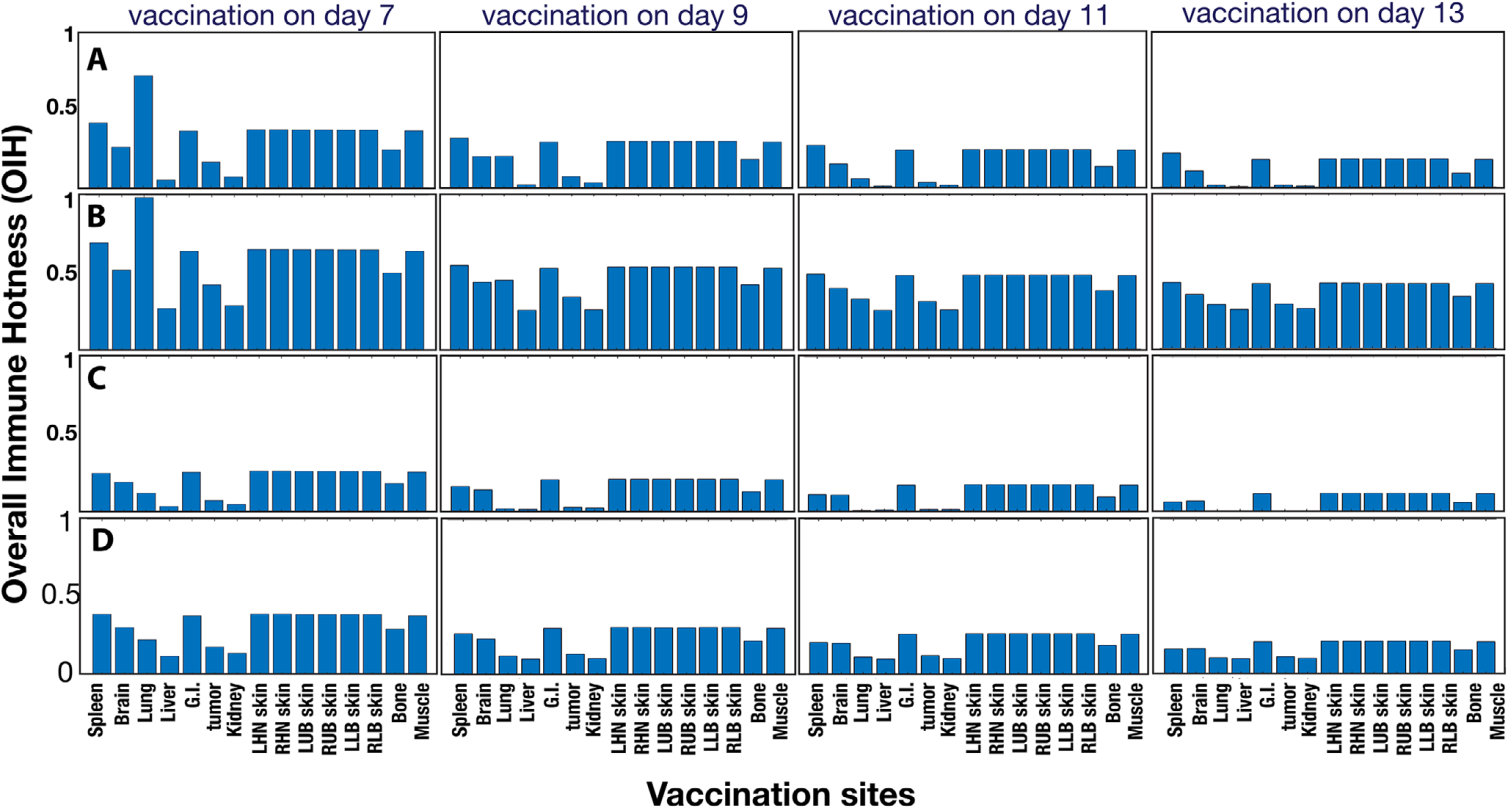
Overall Immune Hotness (OIH) for immunization in different sites at different times post tumor generation. (Row A) Patients with low tumor‐induced antigen and suppressive factor (SF) production rates, both set to −20% relative to baseline (i.e., 0.8 × 10^−3^ min^−1^). (Row B) Patients with a +20% increase in the tumor‐induced antigen production rate relative to Row A, while maintaining the same SF production rate. (Row C) Patients with the same antigen production rate as in Row A but a +20% increase in the SF production rate. (Row D) Patients with +20% increases in both tumor‐induced antigen and SF production rates relative to Row A.

**FIGURE 6:**
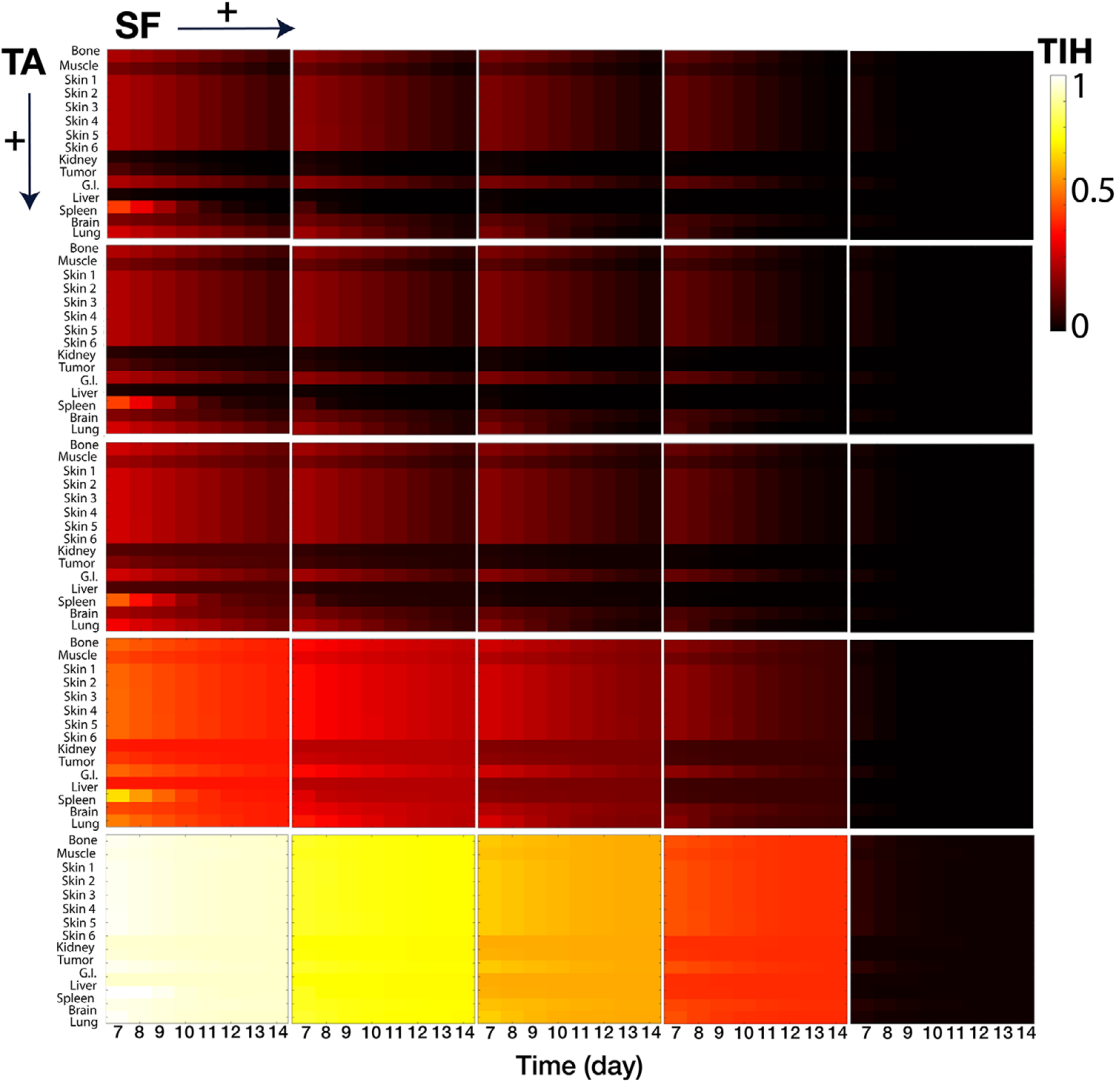
Tumor Immune Hotness (TIH) following immunization at different anatomical sites and time points, under varying levels of tumor‐derived suppressive factors (SF) and tumor antigen (TA). From left to right, the tumor SF production rate increases in 20% increments, corresponding to −40%, −20%, 0%, +20%, and +40% relative to the baseline value (10^−3^ min^−1^); from top to bottom, the TA release rate increases in 20% increments over the same range. Each grid cell represents the area under the curve (AUC) of normalized T cell counts in the tumor microenvironment (TME) over the two weeks following immunization at a given site and time point. Skin regions 1–6 correspond to LHN, RHN, LUB, RUB, LLB, and RLB, respectively.

Biologically, ASO can be interpreted as a measure of the extent and duration of local immune activation within lymphoid tissues and may correspond experimentally to longitudinal measurements of activated or antigen‐specific T cells in draining lymph nodes or spleen. OIH represents the global systemic expansion of activated T cells and is related to experimentally measurable quantities such as circulating antigen‐specific CD8^+^ T‐cell levels in peripheral blood, ELISpot responses, or total activated T‐cell counts measured by flow cytometry. TIH reflects the fraction of activated T cells successfully trafficking to and accumulating within the tumor microenvironment, which is closely associated with experimentally measurable tumor‐infiltrating lymphocyte (TIL) density, CD8^+^ T‐cell infiltration, and clinically observed “hot” versus “cold” tumor phenotypes. Since increased intratumoral CD8^+^ T‐cell infiltration is strongly associated with improved response to immunotherapy, TIH serves as a mechanistic surrogate for anti‐tumor immune efficacy in the model.

For all vaccination routes except intratumoral, ASO is highest in the LNs draining the vaccination site, and the difference between the vaccination LNs and other LNs increases either with higher SF levels (compare patient A with C in Figure 4) or with reduced tumor‐produced antigen (compare patient B with A in Figure 4). In contrast, for intratumoral vaccination, the accumulation of SF in the tumor‐draining LNs (Figure 3) leads to early suppression of either APC activation or T cell activation by activated APCs and results in minimal ASO (Figure 4). In simulations with elevated tumor antigen production, both local and systemic T cell activation are enhanced, leading to higher ASO in vaccination site LNs, other LNs, and spleen (compare patient B with A in Figure 4). In contrast, elevated levels of tumor‐derived SF significantly impair T cell activation in the LNs near the vaccination site and severely reduce activation in most LNs and the spleen—particularly when vaccination occurred within the tumor, which is breast in these simulations (compare patient A with C in Figure 4).

Comparing patients with the same rate of tumor antigen production, those with high SF levels have markedly different responses depending on tumor location and its connectivity to other organs and their respective LNs (patients A and C in Figure 4). For these patients, the farther the vaccination site from the tumor, the more robust the immune response, and intratumoral vaccination elicited a poor response. This is due to the accumulation of SF in tumor draining lymph nodes, which inhibits antigen‐presenting cells (APCs) and directly blocks T cell activation.

As expected, increasing tumor antigen production when SF production is also high (patients D in Figure 4) can mitigate the suppressive effects of the suppressive factor, restoring T cell activation levels to those observed for low SF production and low‐antigen production (patients A and D in Figure 4). This suggests that patients with high antigen levels are less sensitive to both immunosuppression and the location of the vaccination site, particularly when comparing intratumoral and distant vaccination sites. In these patients, the systemic release of tumor‐derived antigens broadly activates lymph nodes throughout the body. As a result, vaccination acts primarily as an additional source of antigen rather than the main driver of immune activation. This effect is especially evident in distal lymph nodes, which are less exposed to suppressive factors (SFs).

These findings suggest that, in patients with tumors producing a high level of suppressive factor, dual‐site vaccination (both intratumoral (similar to when tumor releases antigen itself) and at a distant site) may improve T cell activation. Overall, the temporal dynamics and relative amounts of antigen and SF secretion by the tumor, and the relative anatomic location of the vaccine to the tumor site, dictate the sensitivity of the systemic immune response to vaccination. Therefore, simulating patient‐specific responses based on tumor antigen and SF secretion rates may be necessary to optimize vaccination strategies.

While ASO indicates the best location(s) to apply the vaccine, OIH allows us to analyze immune responses based on vaccination site and timing, reflecting the body's overall capacity to mount an anti‐tumor response. Figure 5 presents OIH values two weeks after immunization (administered on day 7, 9, 11, or 13) for various internal organs and six superficial regions of skin across different patient profiles, categorized by the levels of tumor‐induced antigen and SF. Results show that intradermal and intramuscular vaccinations produce similar OIH levels at each vaccination timing, regardless of site of skin, indicating both are effective delivery routes. In high‐ SF patients, for early vaccination at day 7, the most effective internal vaccination sites (by descending OIH) are: G.I., spleen, bone, brain, lungs, tumor, kidney, and liver (patient C in Figure 5). Late vaccination attenuates this order and equalizes the responses from different vaccination sites. In low‐SF patients (patients A and B)—particularly those with high antigen levels (patients B)—the lung yields the strongest response. These patterns highlight the advantage of immunization strategies based on tumor antigen and SF profiles. Immunization timing also influences vaccination outcomes: repeating simulations at different time points revealed distinct OIH dynamics, underscoring the value of personalized scheduling in cancer immunotherapy.

The ultimate goal of cancer vaccination is to increase the number and efficacy of anti‐cancer immune cells in the tumor. To determine how vaccination affects eT cells in the tumor, we calculate the metric TIH (Figure 6). The heat maps present TIH as a function of vaccination site and timing for patients with variable tumor‐induced antigen and SFs. TIH is highly sensitive to the vaccination site during early stages of tumor development; however, this sensitivity progressively declines over time. The results show that even when vaccination is administered at an early stage of tumor progression, the SF secretion rate can significantly influence TIH, effectively shifting the immune response from fully activated to completely suppressed (as shown from left to right across each row in Figure 6). Furthermore, elevated SF levels diminish the sensitivity of T cell activation to both the timing and site of vaccination (Figure 6).

At low SF levels (in the first column of Figure 6), early vaccination directly within the tumor can still elicit a measurable response, with spleen‐targeted vaccination generating the strongest activation. However, as SF levels rise, the efficacy of spleen‐based vaccination is markedly reduced, and the tumor becomes an ineffective site for inducing immune responses. This is because tumor‐draining LNs (TDLNs) are the first lymph nodes to receive tumor‐derived SF. However, TDLNs also contain high levels of tumor‐associated antigens. Under conditions of low SF, intratumoral vaccination can further elevate antigen levels in the TDLNs, increasing the likelihood of APC and naïve T cell activation. In the spleen, where both antigen and naïve T cells are abundant, vaccination may accelerate T cell activation—provided that the high levels of tumor‐derived SF have not already suppressed immunity.

Increasing tumor antigen levels at any given SF concentration enhances T cell activation and significantly reduces the dependence of immune activation on both vaccination site and timing. Nevertheless, high SF levels continue to suppress overall activation, even in the presence of abundant tumor antigen.

Notably, vaccination in the liver and kidney was ineffective because the injected antigen was rapidly cleared from the tissue through vascular and lymphatic transport and degraded locally, thereby reducing antigen availability for APC activation and subsequent T‐cell priming, even under conditions of low SF level.

Given the wide range of conditions—including tumor antigen levels, suppressive factor concentrations, and variations in vaccination timing and site—large‐scale exploration of virtual patients and vaccination scenarios using the mechanistic model can become computationally demanding. To enable efficient evaluation of these scenarios, we developed a machine learning‐based surrogate framework trained on simulations generated by the mechanistic PBPK/PD model. Specifically, a random forest (RF) algorithm was used to learn the relationship between model inputs and outputs. The predictive performance of the hybrid framework is shown in Figure 7A, where the RF model demonstrated excellent agreement with the mechanistic model (R = 0.99).

**FIGURE 7:**
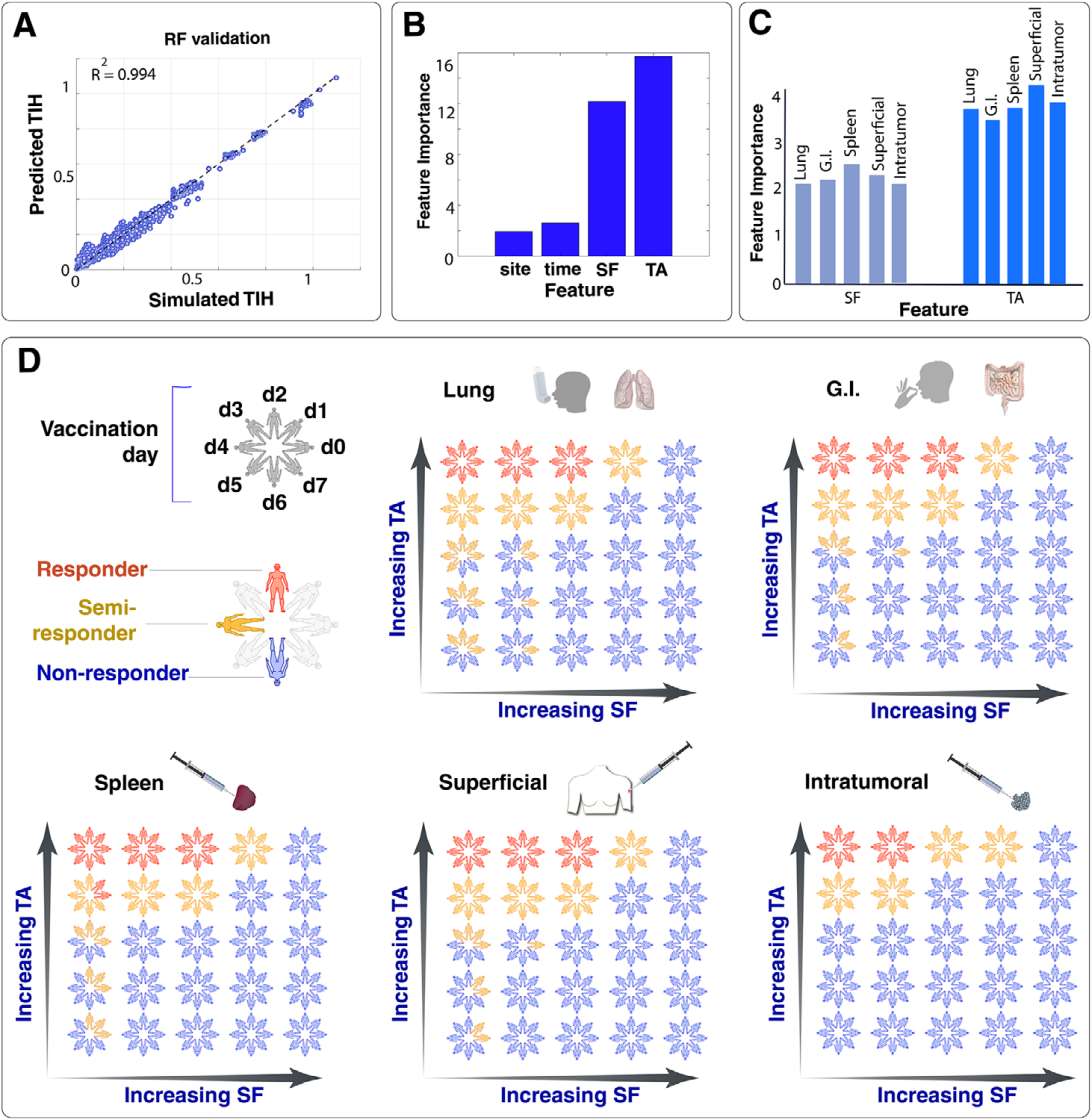
Random Forest (RF) model and feature importance for vaccination outcomes. (A) RF model developed from mechanistic model predictions of TH. (B) Sensitivity of TIH to vaccination site and timing under different SF and TA levels. (C) Site‐specific sensitivity of vaccinations to SF and TA for common sites including lung, G.I., superficial, and intratumoral, as well as spleen, which is unique in its potential for infusion and non‐linear activation due to high immune cell and agent levels. (D) Probability plots for vaccinations in lung, G.I., spleen, superficial, and intratumoral. From bottom to top (TA) and left to right (SF), the production rates increase in 20% increments, corresponding to −40%, −20%, 0%, +20%, and +40% relative to the baseline value of (10^−3^ min^−1^).

Although the mechanistic PBPK/PD model exhibited reasonable computational performance for individual simulations (∼3 min for a 2‐week simulation), the surrogate framework provided additional advantages beyond computational acceleration. In particular, it enabled efficient global analysis of large virtual patient populations and facilitated interpretation of model behavior through feature importance analysis and response clustering. Rather than replacing the mechanistic framework, the surrogate model served as a data‐driven representation of the mechanistic simulations, preserving the dominant relationships between model inputs and outputs while enabling rapid interrogation of the parameter space. These capabilities support future applications in patient stratification, uncertainty analysis, and digital twin development, where thousands of evaluations may be required.

Using the trained RF model, we analyzed the relative influence of vaccination site, vaccination timing, suppressive factor (SF), and tumor antigen (TA) levels on TIH, as shown by the feature importance analysis in Figure 7B. TA was identified as the most influential predictor of TIH, followed by SF, vaccination timing, and vaccination site. Feature importance was further evaluated for selected vaccination routes, including lung, G.I., spleen, superficial, and intratumoral vaccination (Figure 7C). Across all routes, TA remained the dominant predictor, with the greatest influence observed for superficial vaccination and the lowest for G.I. vaccination. The contribution of SF varied by vaccination route, being highest for spleen vaccination, followed by superficial, G.I., lung, and intratumoral vaccination. In contrast, vaccination timing contributed relatively little to TIH variability across the explored parameter space. These results suggest that, for patients with known TA and SF levels, the choice of vaccination site may have a greater impact on systemic immunity outcomes than precise vaccination timing, particularly when the stage of disease is uncertain.

Vaccination timing is a floating parameter dependent on cancer stage, making it a persistent challenge since timely tumor detection is often difficult. To address this, we aimed to estimate the probability of immune response to vaccination over time by repeating vaccination at multiple time points for each patient. In Figure 7D, for each level of SF and TA, simulated patients were vaccinated on eight different days in the lung, G.I., spleen, superficial tissue, and tumor. Using k‐means clustering, responses were grouped into three categories: non‐responder (blue), semi‐responder (yellow), and responder (red). Using the results shown in Figure 4D and the corresponding response colormap, the probability of being classified as a non‐responder, semi‐responder, or responder can be calculated for each vaccination site across patients with varying tumor antigen (TA) and suppressive factor (SF) levels and different vaccination times. For lung vaccination, the probability distribution was 12% responders, 23.5% semi‐responders, and 64.5% non‐responders. For G.I., 12% responders, 19% semi‐responders, and 69% non‐responders. For spleen, 13% responders, 20% semi‐responders, and 67% non‐responders. For superficial, 12% responders, 19% semi‐responders, and 69% non‐responders. For intratumoral, 8% responders, 16% semi‐responders, and 76% non‐responders. Assuming vaccination efficacy for responder = 1, semi‐responder = 0.5, and non‐responder = 0, the overall vaccination efficacy rates for lung, G.I., spleen, superficial, and intratumoral sites were 23.75%, 21.75%, 23%, 21.5%, and 16%, respectively. Therefore, for unknown SF and TA, the suggested descending order of vaccination site efficacy is: lung, spleen, G.I./superficial, and lastly intratumoral. The model and its major immunological components were evaluated at multiple levels using experimental and clinical datasets, including antigen‐dependent T‐cell activation, suppression of APC and T‐cell activation by tumor‐derived factors, and T‐cell responses following therapeutic vaccination. The model showed good agreement with the corresponding experimental and clinical observations. Details of the validation and calibration procedures and quantitative comparisons are provided in the Supplementary Material (Model Validation; Figures S1, S2). Additional analyses were performed to evaluate the effects of local physiological conditions and parameter uncertainty on model predictions. Specifically, we investigated how tumor‐induced changes in local interstitial pressure affect vaccine transport and immune activation across different skin vaccination sites. We also performed a local sensitivity analysis to identify the model parameters with the greatest influence on tumor immune hotness (TIH). These analyses further demonstrate the effects of spatially heterogeneous transport and key immunological parameters on model predictions and are presented in detail in the Supplementary Material (Figures S3, S4).

## 4 Conclusions

Tumor vaccinations hold considerable promise in clinical applications, yet their outcomes remain inconsistent. Critical factors for effective immune activation include: (a) the colocalization of antigen with immature APCs, and (b) the subsequent colocalization of antigen with activated APCs and naïve T cells, which typically occur in secondary lymphoid organs such as lymph nodes and the spleen. However, tumor‐derived suppressive factors can disrupt these activation processes and dampen anti‐tumor immunity. Our simulations reveal that vaccination efficacy is governed not only by the magnitude of systemic immune activation, but also by the spatiotemporal coordination between antigen transport, lymphatic drainage, and suppressive signaling across secondary lymphoid organs. The model predicts that distinct vaccination sites and vaccination timing during tumor progression can generate substantially different immune activation patterns and tumor immune hotness despite similar systemic antigen exposure. While tumor‐driven production of antigen and suppressive mediators plays a central role in initiating immune responses. Importantly, the model predicts that optimal vaccination strategies depend on patient‐specific systemic antigen and suppressive factor levels, suggesting that identical vaccination protocols may produce substantially different immune responses across patients.

The computational framework developed in this study represents a first‐of‐its‐kind multiscale PBPK/PD model that integrates comprehensive systemic circulation with coupled superficial–inner organ networks and a sequential lymph node architecture capturing upstream and downstream lymphatic drainage—features essential for localized immune activation in distinct lymphosomes. The model mechanistically tracks antigen, suppressive factors, naïve and effector T cells, and immature and activated APCs, along with their phenotype‐dependent cytokine release, across both vascular and lymphatic pathways. It also incorporates organ‐specific infiltration dynamics through mechanistic fluxes. Developed using a dynamic matrix‐based approach, the framework can be readily adapted to other species, such as mouse, facilitating translational studies that bridge preclinical and human systems. Unlike conventional PBPK models validated with unrelated species data, this model was verified through a multi‐step, human‐relevant validation process spanning cellular interactions to whole‐body immune responses. This multiscale organization enables mechanistic investigation of organ‐level immune coordination and lymphatic activation dynamics that are difficult to isolate experimentally.

Overall, this mechanistic and human‐validated framework provides quantitative, predictive insights into the competition between immune activation and tumor‐derived suppression, establishing a platform to simulate and optimize personalized vaccination routes, schedules, and timing for improved cancer immunotherapy outcomes. It provides a foundation for digital twin development, which will require (i) patient‐specific parameterization using clinical and omics data, (ii) robust calibration against longitudinal patient datasets, and (iii) prospective validation for predictive use. The current model advances toward this goal by integrating multi‐organ transport, lymph node–resolved activation, and site‐specific vaccination dynamics.

## Author Contributions

**Mohammad R. Nikmaneshi**: conceptualization, investigation, methodology, validation, visualization, writing – review and editing, writing – original draft, project administration, formal analysis, software, data curation, resources. **Timothy P. Padera**: investigation, conceptualization, funding acquisition, writing – review and editing, project administration, supervision, resources. **Lance L. Munn**: funding acquisition, conceptualization, investigation, writing – review and editing, project administration, supervision, resources, methodology.

## Funding

This work was supported by NIH under Grant No. R01CA247441, U01CA261842, and R01CA284603.

## Conflicts of Interest

The authors declare no conflicts of interest.

## Supporting information




**Supporting File 1**: advs78044‐sup‐0001‐SuppMat.docx.

**Supporting File 2**: advs78044‐sup‐0002‐SuppMat.zip.

## Data Availability

The data that support the findings of this study are available from the corresponding author upon reasonable request.
