# Supplementary material for: Optimizing Cancer Vaccinations Using a Physiologically‐Based Pharmacokinetic/Pharmacodynamic (PBPK/PD) Model
Source: Adv Sci (Weinh). 2026 Sep 30:e78044. Online ahead of print. doi: 10.1002/advs.78044 (PMC13627076; doi:10.1002/advs.78044)
Supplement: Supplementary file 1 [file ADVS-9999-e78044-s001.docx]

# Supplementary Material

**Optimizing Cancer Vaccinations Using a Physiologically-Based Pharmacokinetic/Pharmacodynamic (PBPK/PD) Model**

Mohammad R. Nikmaneshi^1^, Timothy P. Padera^1^, Lance Munn.^1,*^

^1^ Department of Radiation Oncology, Massachusetts General Hospital and Harvard Medical School, Boston, MA

02114, USA

* lmunn@mgh.harvard.edu

**CONTENTS**

Supplementary Data and Figures	3

Tumor transport	3

Trafficking through the blood circulation and tumor tissue:	3

Lung	4

Trafficking in the blood and interstitial compartments:	4

Liver, Spleen and Intestine	5

Trafficking in blood vessels and interstitium of liver, intestine and spleen:	5

Muscle, Bone, Brain and Kidney	6

Agent trafficking in blood and interstitium of the j^th^ organ, including skin, muscle, bone, brain and kidney:	6

Skin; Superficial Network	7

In this model, to allow testing of vaccine injections at different skin sites, the superficial skin network was divided into six zones: Left Head and Neck (LHN), Right Head and Neck (RHN), Left Upper Body (LUB), Right Upper Body (RUB), Left Lower Body (LLB), and Right Lower Body (RLB).	7

Agents trafficking in blood and interstitium of the j^th^ part of skin, including LHN (j=1), RHN (j=2), LUB (j=3), RUB (j=4), LLB (j=5), RLB (j=6):	7

LNs; ULNs and DLNs (all LNs except abdominal LNs)	7

Trafficking to the draining lymph nodes; upstream LNs (ULNs) and downstream LNs (DLNs)	7

**Agents trafficking in the abdominal LNs (LN_Abd)**	9

for ULNs, the equations of these LNs are different because of different connections of these LNs compared to other LNs (see the computational chart in Fig.1).	9

Heart	10

a) Agents recirculation in arterial and venous blood flows	10

b) Mass conservation equations	10

Tumor compartment; reactions, growth and angiogenesis	10

a)	Tumor growth and angiogenesis:	10

b)	Tumor-induced suppressive factors (SFs):	12

c)	Antigen and vaccination model:	12

d)	Inflammatory Cytokines:	12

e)	T cell-induced activation Cytokines (IL-2):	12

f)	Immature APCs (imAPC) and activated APCs (aAPCs):	13

g)	Naïve T cells (nTs) and activated effector T cells (eTs):	13

Transvascular and Lymphatic Transport of Agents	14

Model validation	16

Effect of tumor-induced local pressure on vaccination on different parts of skin	19

Sensitivity Analysis to Identify Influential Parameters	20

Table S. Model parameters	22

## **Supplementary Data and Figures**

**Compartmental PKPD model:**

We used similar approach to our previous work^1^ to develop the compartmental pharmacokinetic-pharmacodynamics (PKPD) model, but this time with more physiological and anatomical details. To develop the mass transport, we used the mass balance for each agents in different parts of each compartments including blood flow, transmural flow, lymphatic flow by considering details of mass and fluid flow across blood vessels, interstitium and collecting vessels.

We present the model by each specific organ and its parts including blood, lymphatic flow, tissue, and lymph nodes (LNs). Notably, in the compartmental model, the tumor can be hosted by any of the organs. Here we simulate a tumor growing in the breast.

In this model, molecular and cellular agents follow different states during transvascular transport from the vascular compartment into the interstitium. Cellular agents transition through three states: bloodstream, captured, and interstitial. In contrast, molecular agents do not undergo a captured state; therefore, their transvascular transport is described by two states: bloodstream and interstitial. However, in the simulations, we assume that the captured-cell state is quasi-steady. Consequently, the three equations describing cellular transport are reduced to two equations, with a modified effective transmigration rate, as explained in the section “**Transvascular and Lymphatic Transport of Agents**”.

### **Tumor transport**

#### Trafficking through the blood circulation and tumor tissue:

The mass balance for i^th^ agent including cells and molecules in the tumor bloodstream (*ci^b^_tumor_*) is:

$\frac{d{ci}_{tumor}^{b}}{dt}=\left( \begin{aligned} &f_{tumor}Q_{tumor}{ci}_{Heart}^{b}-(f_{tumor}Q_{tumor}-L_{tumor}){ci}_{tumor}^{b} \\ &-a_{tumor}{ci}_{tumor}^{b}V_{tumor}^{b} \\ &+d_{tumor}{ci}_{tumor}^{c}V_{tumor}^{b} \end{aligned} \right)/V_{tumor}^{b}$

(1a)

where the first term is the flow of agent from the arterial blood into the tumor; the second term is the flow of agent out of the tumor via the venous blood flow that equals inlet arterial flow minus filtered interstitial flow/lymphatic flow (*f Q - L*); the third term represents the binding of agents to the tumor endothelium, and the last term is the detachment of cells/agents from the endothelium. The two last terms are only for cells including antigen presenting cells (APCs) and T cells. In the normal organs, attachment and detachment rates (*a* and *d*) are assumed to be constant. In the tumor compartment, we assume that the vascular attachment and detachment rates (*a* and *d*) of cells depends on tumor size (Table. S).

The mass balance for the cells captured on the vessel wall (*ci^c^_tumor_*) is as equation (1b), however the molecules do not have the captured phase and directly convert form blood born to interstitial agents by equation (1c):

$$\frac{d{ci}_{tumor}^{c}}{dt}=\left( a_{tumor}{ci}_{tumor}^{b}V_{tumor}^{b}-d_{tumor}{ci}_{tumor}^{c}V_{tumor}^{b}-J_{ci,tumor}{ci}_{tumor}^{c}V_{tumor}^{b}-{J_{d}}_{ci,tumor} \right)/V_{tumor}^{b}$$

(1b)

Where the first term is the binding of cells to the vessel wall, the second term is the detachment and the third term is transmigration. The last term is a diffusion flux (${J_{d}}_{ci,tumor}$), consistent with the classical Stokes–Einstein / Kedem–Katchalsky transport framework, and is **only** applicable to molecular agents. Cellular agents (T cells and APCs here) transmigrate solely via the transmigration flux, represented by the third term ($J_{ci,\mathrm{tumor}}$), using the parameter values reported in the Supplementary Material of our previous study ^1^.

The mass balance for cells in the tumor tissue (*ci^ins^_tumor_*) is:

$$\frac{d{ci}_{tumor}^{ins}}{dt}=\left( \begin{aligned} &J_{ci,tumor}{ci}_{tumor}^{c}V_{tumor}^{b}+{J_{d}}_{ci,tumor}-L_{tumor}{ci}_{tumor}^{ins}fi_{ci,tumor} \\ &+{R_{ci}}_{tumor}^{ins} V_{tumor}^{ins} \end{aligned} \right)/V_{tumor}^{ins}$$

(1c)

Where the first term is agent transmigration into the tumor from blood vessels, the second term is diffusion flux, the third term is cell entry into lymphatic vessels; the last term represents the specific reactions for each agents in tumor interstitium. *J* represents the transmigration(for cells)/convection(for molecules) flux and $J_{d}$ represents the diffusion flux, calculated using the modified Starling’s law developed in our previous work^2^. It is a function of the particle size, vessel wall porosity, transmural pressure, and oncotic pressure^2^. It relies on both convection (cells and molecules) and diffusion (molecules) across the vessel wall^2^. Details of this model are presented in the section " **Transvascular and Lymphatic Transport of Agents**." Note that the diffusion and convection fluxes are **only** applicable to molecular agents. Cellular agents (T cells and APCs here) transmigrate solely via the transmigration flux, represented by the third term ($J_{ci,\mathrm{tumor}}$), using the parameter values reported in the Supplementary Material of our previous study ^1^.

### **Lung**

#### Trafficking in the blood and interstitial compartments:

In other organs, such as lung, agents ($ci$) enter and leave via the bloodstream; Cellular agents (here, T cells and APCs) can also attach to and detach from the vessel wall:

$\frac{d{ci}_{lung}^{b}}{dt}=\left( \begin{aligned} &Q_{lung}{ci}_{Heart}^{v}-\left( Q_{lung}-L_{lung} \right){ci}_{lung}^{b} \\ &-a_{lung} {ci}_{lung}^{b}V_{lung}^{b} \\ &+d_{lung} {ci}_{lung}^{c}V_{lung}^{b} \end{aligned} \right)/V_{lung}^{b}$

(2a)

The first term is the flow of agents from the venous flow into the lung, the second term is the agents leaving the lung via outlet of blood flow, the last terms are attachment and detachment rates of cells from and to lung vessels.

The agents can extravasate into the interstitial compartment:

$$\frac{d{ci}_{lung}^{c}}{dt}=\left( a_{lung} {ci}_{lung}^{b}V_{lung}^{b}-d_{lung} {ci}_{lung}^{c}V_{lung}^{b}-J_{ci,lung}{ci}_{lung}^{c}V_{lung}^{b}-{J_{d}}_{ci,lung} \right)/V_{lung}^{b}$$

(2b)

In this equation, the first term is the attachement rate of agents to the lung vessels, the second term is the detachment rate of agents from the lung vessels, and the two last terms are the transmigration (for cells)/convection (for molecules) rate of agents from vessels to tissue and the diffusion rate of agents across the vessel wall. Note that the diffusion and convection fluxes are **only** applicable to molecular agents. Cellular agents (T cells and APCs here) transmigrate solely via the transmigration flux, represented by the third term ($J_{ci,\mathrm{tumor}}$), using the parameter values reported in the Supplementary Material of our previous study ^1^.

The agents enter the lung tissue by transmigration, and leave via the lymphatic system:

$$\frac{d{ci}_{lung}^{ins}}{dt}=\left( J_{ci,lung}{ci}_{lung}^{c}V_{lung}^{b}+{J_{d}}_{ci,lung}-L_{lung}{ci}_{lung}^{ins}fi_{ci,lung} \right)/V_{lung}^{ins}$$

(2c)

Where, the first term is the transmigration(for cells)/convection (for molecules) rate of agents, the second term is diffusion flux (only for molecules), and the last term is the agents flow out of tissue part of lung.

### **Liver, Spleen and Intestine**

#### Trafficking in blood vessels and interstitium of liver, intestine and spleen:

These organs have a connected network of LNs. We assume a common LN region for the abdominal LN to show this connectivity. The upstream blood flow can enter intestine, spleen and liver and after plasma filtration, the interstitial plasma flows of these three organs can collect into their common LN region.

*For the Liver:*

| $\frac{d{ci}_{liver}^{b}}{dt}=\left( \begin{aligned} &f_{liver}(Q_{int estine}-L_{int estine}){ci}_{int estine}^{b}+f_{liver}(Q_{spleen}-L_{spleen}){ci}_{spleen}^{b} \\ &+f_{liver}(Q_{liver}-Q_{int estine}-Q_{spleen}+L_{int estine}+L_{spleen}){ci}_{Heart}^{a} \\ &-(f_{liver}Q_{liver}-L_{liver}){ci}_{liver}^{b} \\ &-a_{liver}{ci}_{liver}^{b}V_{liver}^{b} \\ &+d_{liver}{ci}_{liver}^{c}V_{liver}^{b} \end{aligned} \right)/V_{liver}^{b}$ | | | (3a) |
| --- | --- | --- | --- |
| $\frac{d{ci}_{liver}^{c}}{dt}=\left( a_{liver}{ci}_{liver}^{b}V_{liver}^{b}-d_{liver}{ci}_{liver}^{c}V_{liver}^{b}-J_{ci,liver}{ci}_{liver}^{c}V_{liver}^{b}-{J_{d}}_{ci,liver} \right)/V_{liver}^{b}$ | (3b) | | |
| $\frac{d{ci}_{liver}^{ins}}{dt}=\left( J_{ci,liver}{ci}_{liver}^{c}V_{liver}^{b}+{J_{d}}_{ci,liver}-L_{liver}{ci}_{liver}^{ins}fi_{ci,liver} \right)/V_{liver}^{ins}$ | | (3c) | |

In Eq. 3a, the first and second terms are the flow of agents from the outlet blood flows of intestine and spleen into the liver, the third term is the flow of agents from the arterial blood into the liver; the fourth term is the flow of agents out of the liver; the two last terms represent the binding of agents to the liver endothelium and the detachment of agents from the endothelium. In Eqs. 3b and 3c, the terms with the parameter *J* are transmigration (for cells)/convection (for molecules) rates of agents into the liver interstitium and $J_{d}$ is diffusion flux (only for molecules).

*For the Spleen:*

| $\frac{d{ci}_{spleen}^{b}}{dt}=\left( \begin{aligned} &Q_{spleen}{ci}_{Heart}^{v}-(Q_{spleen}-L_{spleen}){ci}_{spleen}^{b} \\ &-a_{spleen}{ci}_{spleen}^{b}V_{spleen}^{b} \\ &+d_{spleen}{ci}_{spleen}^{c}V_{spleen}^{b} \end{aligned} \right)/V_{spleen}^{b}$ | (3d) |
| --- | --- |
| $\frac{d{ci}_{spleen}^{c}}{dt}=\left( a_{spleen}{ci}_{spleen}^{b}V_{spleen}^{b}-d_{spleen}{ci}_{spleen}^{c}V_{spleen}^{b}-J_{ci,spleen}{ci}_{spleen}^{c}V_{spleen}^{b}-{J_{d}}_{ci,spleen} \right)/V_{spleen}^{b}$ | (3e) |
| $\frac{d{ci}_{spleen}^{ins}}{dt}=\left( J_{ci,spleen}{ci}_{spleen}^{c}V_{spleen}^{b}+{J_{d}}_{ci,spleen}-L_{spleen}{ci}_{spleen}^{ins}fi_{ci,spleen} \right)/V_{liver}^{ins}$ | (3f) |

In Eq. 3d, the first term is the flow of agents from the arterial blood into the spleen; the second term is the flow of agents out of the spleen; the third term represents the binding of agents to the spleen endothelium, and the last term is the detachment of cells from the endothelium. In Eqs. 3e and 3f, the terms with *J* are transmigration (for cells)/convection (for molecules) rates of agents into the spleen interstitium and $J_{d}$ is diffusion flux (only for molecules). The last term in Eq.3f is the drainage of agents by filtration from the spleen interstitium to lymphatic vessels.

*For the Intestine:*

| $\frac{d{ci}_{int estine}^{b}}{dt}=\left( \begin{aligned} &Q_{int estine}{ci}_{Heart}^{v}-\left( Q_{int estine}-L_{int estine} \right) {ci}_{int estine}^{b} \\ &-a_{int estine}{ci}_{int estine}^{b}V_{int estine}^{b} \\ &+d_{int estine}{ci}_{int estine}^{c}V_{int estine}^{b} \end{aligned} \right)/V_{int estine}^{b}$ | (3g) |
| --- | --- |
| $\frac{d{ci}_{int estine}^{c}}{dt}=\left( \begin{aligned} &a_{int estine}{ci}_{int estine}^{b}V_{int estine}^{b}-d_{int estine} {ci}_{int estine}^{c}V_{int estine}^{b} \\ &-J_{ci,int estine} {ci}_{int estine}^{c}V_{int estine}^{b}-{J_{d}}_{ci,intestine} \end{aligned} \right)/V_{int estine}^{b}$ | (3h) |
| $\frac{d{ci}_{int estine}^{ins}}{dt}=\left( J_{ci,int estine}{ci}_{int estine}^{c}V_{int estine}^{b}+{J_{d}}_{ci,intestine}-L_{int estine}{ci}_{int estine}^{ins}fi_{ci,int estine} \right)/V_{int estine}^{ins}$ | (3l) |

In Eq. 3g, the first term is the flow of agents from the arterial blood into the intestine; the second term is the flow of agents out of the intestine; the third term represents the binding of agents to the intestine endothelium, and the last term is the detachment of agents from the intestine endothelium. In Eqs. 3h and 3l, the terms with *J* are transmigration (for cells)/convection (for molecules) rates of agents into the intestine interstitium and $J_{d}$ is diffusion flux (only for molecules). The last term in Eq.3l is the drainage of agents by filtration from the intestine interstitium to lymphatic vessels. Again, only cells include attachment and detachment terms before transmigrating under the influence of *J*, whereas molecules directly transmigrate according to their respective *J* and $J_{d}$.

### **Muscle, Bone, Brain and Kidney**

#### Agent trafficking in blood and interstitium of the j^th^ organ, including skin, muscle, bone, brain and kidney:

$\frac{d{ci}_{j}^{b}}{dt}=\left( \begin{aligned} &f_{j}Q_{j}{ci}_{Heart}^{a}-(f_{j}Q_{j}-L_{j}){ci}_{j}^{b} \\ &-{ai}_{j}{ci}_{j}^{b} V_{j}^{b} \\ &+{di}_{j}{ci}_{j}^{c} V_{j}^{b} \end{aligned} \right)/V_{j}^{b}$

(4a)

$$\frac{d{ci}_{j}^{c}}{dt}=\left( {ai}_{j}{ci}_{j}^{b} V_{j}^{b}-{di}_{j}{ci}_{j}^{c} V_{j}^{b}-J_{ci,j}{ci}_{j}^{c}V_{j}^{b}-{J_{d}}_{ci,j} \right)/V_{j}^{b}$$

(4b)

$$\frac{d{ci}_{j}^{ins}}{dt}=\left( J_{ci,j}{ci}_{j}^{c}V_{j}^{b}+{J_{d}}_{ci,j}-L_{j}{ci}_{j}^{ins}fi_{ci,j} \right)/V_{j}^{ins}$$

(4c)

In Eq. 4a, the first term is the flow of agents from the arterial blood into the *j^th^* organ; the second term is the flow of agents out of the *j^th^* organ; the third term represents the binding of agents to the endothelium of *j^th^* organ, and the last term is the detachment of cells from the endothelium. In Eqs. 4b and c, the terms with *J* are transmigration (for cells)/convection (for molecules) rates of agents into the tissue interstitium and $J_{d}$ is diffusion flux (only for molecules), and the term with *L* is the outflow of T cells and APCs via lymphatic flow:

### **Skin; Superficial Network**

#### In this model, to allow testing of vaccine injections at different skin sites, the superficial skin network was divided into six zones: Left Head and Neck (LHN), Right Head and Neck (RHN), Left Upper Body (LUB), Right Upper Body (RUB), Left Lower Body (LLB), and Right Lower Body (RLB).

#### Agents trafficking in blood and interstitium of the j^th^ part of skin, including LHN (j=1), RHN (j=2), LUB (j=3), RUB (j=4), LLB (j=5), RLB (j=6):

$\frac{d{ci}_{j}^{b}}{dt}=\left( \begin{aligned} &f_{j}Q_{j}{ci}_{Heart}^{a}-(f_{j}Q_{j}-L_{j}){ci}_{j}^{b} \\ &-{ai}_{j}{ci}_{j}^{b}V_{j}^{b} \\ &+{bi}_{j}{ci}_{j}^{b}V_{j}^{b} \end{aligned} \right)/V_{j}^{b}$

(5a)

$$\frac{d{ci}_{j}^{c}}{dt}=\left( {ai}_{j}{ci}_{j}^{b}V_{j}^{b}-{bi}_{j}{ci}_{j}^{b}V_{j}^{b}-J_{ci,j}{ci}_{j}^{c}V_{j}^{b}-{J_{d}}_{ci,j} \right)/V_{j}^{b}$$

(5b)

$$\frac{d{ci}_{j}^{ins}}{dt}=\frac{\left( J_{ci,j}{ci}_{j}^{c}V_{j}^{b}+{J_{d}}_{ci,j}-L_{j}{ci}_{j}^{ins}fi_{ci,j}-\sum_{k\neq j}^{{k=N}_{j,k}} {f_{jk} L}_{j} {ci}_{j}^{ins}+\sum_{k\neq j}^{{k=N}_{j,k}} {f_{kj} L}_{k} {ci}_{k}^{ins} \right)}{V_{j}^{ins}}$$

$$, j=1 \left( LHN \right) ;N_{j,k}=2,3,4$$

$$, j=2 \left( RHN \right);N_{j,k}=1,3,4$$

$$, j=3 \left( LUB \right);N_{j,k}=1,2,4,5,6$$

$$, j=4 \left( RUB \right);N_{j,k}=1,2,3,5,6$$

$$, j=5 \left( LLB \right);N_{j,k}=3,4,6$$

$$, j=6 \left( RLB \right);N_{j,k}=3,4,5$$

(5c)

In Eq. 5a, the first term is the flow of agents from the arterial blood into the *j^th^* part of skin; the second term is the flow of agents out of the *j^th^* part of skin; the third term represents the binding of agents (cells) to the endothelium of the *j^th^* part of skin, and the last term is the detachment of cells from the endothelium. In Eqs. 5b and c, the terms with *J* are transmigration (for cells)/convection (for molecules) rates of agents into the tissue interstitium and $J_{d}$ is diffusion flux (only for molecules), and the term with *L* is the outflow of agents via lymphatic flow.

### **LNs; ULNs and DLNs (all LNs except abdominal LNs)**

#### Trafficking to the draining lymph nodes; upstream LNs (ULNs) and downstream LNs (DLNs)

Molecules such as antigen, SFs, and cytokines can enter LNs through blood stream and transfer to the LN interstitium based on their size, filtration velocity, porosity and permeability of vessel walls, hydrostatic and oncotic pressure gradients, all impacting through a *J* parameter defined in our previous tumor model^2^. T cells enter the LN via the blood stream; they can exit with the blood flow, and the naïve T cell can be captured; however, effector T cells cannot be captured by attaching to the HEVs in the node ($J_{eT,LN}$ =0).

Note, in these notations, index *j* reads ID of LNs related to each organ; ULNj and DLNj are ULN and DLN of the *j^th^* organ. Subindex *b* means blood and ins means interstitial agent, *ci* means concentration of the *i^th^* agent.

$\frac{d{ci}_{ULNj}^{b}}{dt}=\left( \begin{aligned} &Q_{ULNj} {ci}_{heart}^{b} \\ &-\left( Q_{ULNj}-L_{ULNj} \right) {ci}_{ULNj}^{b}-J_{ci,ULNj} {ci}_{ULNj}^{b} V_{ULNj}^{b}-{J_{d}}_{ci,ULN_{j}} \end{aligned} \right)/V_{ULNj}^{b}$

$$for all LNs except lung: Q_{ULNj}= \left( 1-f_{j} \right) Q_{j}$$

$$for lung: Q_{ULN, lung}= \left( 1-f_{lung} \right) (Q_{j}-L_{j})$$

(6a)

Agents including cells and molecules enter from upstream tissue via lymph flow from afferent ports and can exit from efferent ports:

$$\frac{d{ci}_{ULNj}^{ins}}{dt}=\left( \begin{aligned} &{R_{ci}}_{ULNj}^{ins} V_{ULNj}^{ins} \\ +J_{ci,ULNj} {ci}_{ULNj}^{b} V_{ULNj}^{b}+{J_{d}}_{ci,ULN_{j}} \\ &+\left( 1-ff_{j} \right) L_{j} {ci}_{j}^{ins} fi_{ci,j} \\ &-\left( \left( 1-ff_{j} \right) L_{j}+L_{ULNj} \right)\times\left( fi_{ci,ULNj} \right){ci}_{ULNj}^{ins} \\ & \\ & \end{aligned} \right)/V_{ULNj}^{ins}$$

(6b)

where the first term is the reaction term presented specifically for each agent in the next section. For instance, for eTs, R is proliferation rate of eT cells in the activated LNs. The second and third terms are transmigration/convection of agents from vessels into the interstitium of ULNs and the diffusion flux of molecules, respectively. The forth term of Eq.6b is agents flow from lymphatic flow of tissue into interstitium of LNs, and the last term is the fraction of agents that bypasses the LN and exits at the efferent lymphatic vessel.

The proliferation rate of cells is a function of antigen concentration in the LN, IL-2 presence in the LN, and the level of activated cells in the LN. The same set of equations for DLN, but it has one lymphatic flow from ULN.

$\frac{d{ci}_{DLNj}^{b}}{dt}=\left( \begin{aligned} &Q_{DLNj} {ci}_{heart}^{b} \\ &-\left( Q_{DLNj}-L_{DLNj} \right) {ci}_{DLNj}^{b}-J_{ci,DLNj} {ci}_{DLNj}^{c} V_{DLNj}^{b}-{J_{d}}_{ci,DLN_{j}} \end{aligned} \right)/V_{DLNj}^{b}$

$$for all LNs except lung: Q_{DLNj}= \left( 1-f_{j} \right) Q_{j}$$

$$for lung: Q_{DLN, lung}= \left( 1-f_{lung} \right) (Q_{j}-L_{j})$$

(6c)

Agents enter from upstream tissue via lymph flow from afferent ports and can exit from efferent ports:

$$\frac{d{ci}_{DLNj}^{ins}}{dt}=\left( \begin{aligned} &{R_{ci}}_{LN}^{ins} V_{DLNj}^{ins} \\ +J_{ci,DLNj} {ci}_{DLNj}^{c} V_{DLNj}^{b}+{J_{d}}_{ci,DLN_{j}} \\ +\left( 1-f{f^{'}}_{j} \right)\left( \left( 1-ff_{j} \right) L_{j}+L_{ULNj} \right)\times\left( fi_{ci,ULNj} \right){ci}_{ULNj}^{ins} \\ \\ &-\left( \left( 1-f{f^{'}}_{j} \right)\left( \left( 1-ff_{j} \right) L_{j}+L_{ULNj} \right)+L_{DLNj} \right)\times( fi_{ci,DLNj}){ci}_{DLNj}^{ins} \\ & \\ & \end{aligned} \right)/V_{DLNj}^{ins}$$

(6d)

#### **Agents trafficking in the abdominal LNs (LN_Abd)**

#### for ULNs, the equations of these LNs are different because of different connections of these LNs compared to other LNs (see the computational chart in Fig.1).

| $\frac{d{ci}_{ULN\_Abd}^{b}}{dt}=\left( \begin{aligned} &(1-f_{liver})(Q_{int estine}-L_{int estine}){ci}_{int estine}^{b} \\ &+(1-f_{liver})(Q_{spleen}-L_{spleen}){ci}_{spleen}^{b} \\ &+(1-f_{liver})(Q_{liver}-Q_{int estine}-Q_{spleen}+L_{int estine}+L_{spleen}){ci}_{Heart}^{a} \\ -\left( \left( 1-f_{liver} \right)Q_{liver}-L_{ULNj\_Abd} \right){ci}_{ULN\_Abd}^{b} \\ -J_{ci,ULN_{Abd}} {ci}_{ULN_{Abd}}^{b} V_{ULN_{Abd}}^{b}-{J_{d}}_{ci,ULN_{Abd}} \\ & \end{aligned} \right)/V_{ULN\_Abd}^{b}$ | (7(7a) |
| --- | --- |

Where the first term is the agents flow in LNs via blood flow of the intestine, the second term is the agnets flow in LNs via blood flow of the spleen, the third term is the agents flow from outflow of blood into the liver, and the forth term is the fraction of agents that bypasses the LN and exits at the efferent lymphatic vessel. The last two terms are transmigration (for cells)/convection (for molecules) rates of agents into the tissue interstitium and diffusion flux (only for molecules), respectively.

$$\frac{d{ci}_{ULN\_Abd}^{ins}}{dt}=\left( \begin{aligned} &{R_{ci}}_{ULN\_Abd}^{ins} V_{ULN\_Abd}^{ins} \\ +J_{ci,ULN\_Abd} {ci}_{ULN\_Abd}^{b} V_{ULN\_Abd}^{b}+{J_{d}}_{ci,ULN_{Abd}} \\ +\left( 1-ff_{Abd} \right)L_{liver} {ci}_{liver}^{ins}fi_{ci,liver} \\ +(1-ff_{Abd})L_{int estine}{ci}_{int estine}^{ins}fi_{ci,int estine} \\ &+(1-ff_{Abd})L_{spleen}{ci}_{spleen}^{ins}fi_{ci,spleen} \\ &-\left( \left( 1-ff_{Abd} \right)(L_{liver}+L_{int estine}{+L}_{spleen})+L_{ULN\_Abd} \right) \\ &\times\left( fi_{ci,ULN_{Abd}} \right){ci}_{ULN\_Abd}^{ins} \end{aligned} \right)/V_{ULN\_Abd}^{ins}$$

(7b)

where the first term is the proliferation rate of agents (only cells) in antigen-positive LNs. The second and third terms are transmigration (for cells)/convection (for molecules) rates of agents into the ULNs interstitium and diffusion flux (only for molecules), respectively. The fourth, fifth, and sixth terms are agents flow from lymphatic flow of tissue into LNs, and the last term is the fraction of agents that bypasses the LN and exits at the efferent lymphatic vessel. The proliferation rate of agents (only cells) is a function of antigen concentration in the LN, IL-2 presence in the LN, and the level of activated cells in the LN. The same blood flow paths for DLNs, except their connection to ULNs follows the same trend as common DLNs in equation 6d.

### **Heart**

#### a) Agents recirculation in arterial and venous blood flows

$\frac{d{ci}_{Heart}^{a}}{dt}=\left( \begin{aligned} &f_{lung} \left( Q_{lung}-L_{lung} \right) {ci}_{lung}^{b} \\ &-(Q_{liver}+L_{spleen}+L_{int estine}+Q_{kidney}+Q_{tumor} \\ &+\sum Q_{skin}+Q_{muscle}+Q_{bone}+Q_{brain}) {ci}_{Heart}^{a} \end{aligned} \right)/V_{Heart}^{a}$

(8a)

Where the first term is the agent flow from lung to left ventricle of heart and the second term is the agent flow leaving the left ventricle to distribute among different organs.

$\frac{d{ci}_{Heart}^{v}}{dt}=\left( \sum_{j}^{N} {(Q-L)}_{outj}{ci}_{j}^{b}+L_{outj}{ci}_{j}^{ins} fi_{ci,j})-Q_{lung}{ci}_{Heart}^{v} \right)/V_{Heart}^{a}$

(8b)

Where the first term is flow of agents into venous flow of heart and the second term is collected agents flow leaving the heart via right ventricle flow (inflow of lung). Note that, based on mass conservation, ${(Q-L)}_{outj}$ and $L_{outj}$ for different tissues and their LNs are calculated as below:

| $for lung: {(Q-L)}_{outj}= Q_{j}-L_{j}$  $for other tissues : {(Q-L)}_{outj}= f_{j}Q_{j}-L_{j}$ | (8b*) |
| --- | --- |

$$for lung LNs : {(Q-L)}_{outj}=\left( \left( 1-f_{j} \right) \left( Q_{j}-L_{j} \right)-L_{LNs,j} \right)$$

$for other LNs : {(Q-L)}_{outj}=\left( \left( 1-f_{j} \right) Q_{j}-L_{LNs,j} \right)$

| $for all organs: L_{outj}= ff_{i} L_{j}$ | (8b*) |
| --- | --- |

$$for liver ULNs : L_{outj}=f{f^{'}}_{Abd}\left( \left( 1-ff_{Abd} \right)\left( L_{liver}+L_{int estine}{+L}_{spleen} \right)+L_{ULN_{Abd}} \right)$$

$$for liver DLNs : L_{outj}=\left( 1-f{f^{'}}_{Abd} \right)\left( \left( 1-ff_{Abd} \right)\left( L_{liver}+L_{int estine}{+L}_{spleen} \right)+L_{ULN_{Abd}} \right)+L_{DLN_{Abd}}$$

$$for other ULNs : L_{outj}=f{f^{'}}_{j} \left( \left( 1-ff_{j} \right)\left( L_{j} \right)+L_{ULNj} \right)$$

$for other DLNs : L_{outj}=\left( 1-f{f^{'}}_{j} \right)\left( \left( 1-ff_{j} \right)\left( L_{j} \right)+L_{ULNj} \right)$+$L_{DLNj}$

#### b) Mass conservation equations

$f_{lung}Q_{lung}=f_{lung}L_{lung}+Q_{heart-to-liver}+Q_{spleen}+Q_{int estine}+Q_{kidney}+Q_{tumor}+\sum Q_{skin}+Q_{muscle}+Q_{bone}+Q_{brain}$

(8c)

$Q_{liver}=Q_{heart-to-liver}+(Q_{spleen}-L_{spleen})+(Q_{int estine}-L_{int estine})$

(8d)

### **Tumor compartment; reactions, growth and angiogenesis**

#### a) Tumor growth and angiogenesis:

cancer cells proliferate, are bound by T cells ($j :eT cells \to c_{j}=c_{eT}$) and can de-conjugate and return to the active cell population. In this model, cancer cells can be static and grow in tumor tissue or circulating to model metastatic based on their accumulation in secondary places. Here, we assume those are static in tumor tissue.

The mass balance for active cancer cells,$C$, is:

| $\frac{dC}{dt}=\left( \begin{aligned} &g_{v}\left( \frac{v}{C+v} \right)C+g_{av}\left( \frac{C}{C+\lambda_{a0}} \right)+\left( k_{-1}+k_{2}\left( 1-p_{r} \right) \right)\alpha_{eT,C} \\ &-k_{1}\left( 1+\beta_{v}\frac{v}{v+\alpha_{v}} \right){c_{eT}}_{tumor}^{ins}C \end{aligned} \right)$ | | (9a) |
| --- | --- | --- |
| Where the first term is the vascularized tumor growth as a function of angiogenic vessels, *v*, and active cancer cells, $C$, the second term is avascularized tumor growth in the form of Michaelis–Menten model, the third term is the sumation of the de-conjugation rate of cancer cells from tumor/T cell complexes (*k_-1_ α_T,C_*), and the rate at which cancer cells survive T cell interactions and return to the cancer cell compartment, (*k_2_* (1*-p_r_*) *α_T,C_*). The latter is complementary to the immunogenic cell death (ICD) rate, (*k_2_* (*p_r_*) *α_T,C_*), which is negligible in the present study since (*p_r_* is ~0). The last term is the generation of tumor/T cell complexes with the help of angiogenic vessels. Indeed, the presence of vessels allows T cells to access a larger portion of the tumor compared to avascular tumors.  The mass balance for cancer-immune cell conjugates $\alpha_{T,C}$ is: | | |
| $\frac{d\alpha_{T,C}}{dt}=\left( k_{1}(1+\beta_{v}\frac{v}{v+\alpha_{v}}){c_{eT}}_{tumor}^{ins}C-\left( k_{-1}+k_{2} \right)\alpha_{eT,C} \right)$ | (9b) | |

Where the first term is production rate of complex formation as a function of angiogenic vessels that can help eT cells penetrate deeper regions of cancer and make complex with inner cells, and the second term is reduction rate of cancer/T cell complex immediately after formation with reversibility rate (*k_-1_*) and after doing reaction with T cells with rate *k_2_*_._

To model angiogenesis, we assumed that cancer cells secrete vascular endothelial growth factors (VEGF) that can stimulate endothelial cells to start angiogenesis. The concentration of tumor-induced VEGF, ***c_vegf_*,** is given by:

$\frac{dc_{vegf}}{dt}=\left( \eta_{ag}(C+\alpha_{T,C})-\varepsilon_{vegf}c_{vegf} \right)$

(9c)

where the first term is the production rate of VEGF by cancer cells (both de-conjugated and conjugated with T cells), and the second term is the natural decay of VEGF.

To model angiogenesis mechanism for vascular density, we developed equation (9d) based on VEGF concentration. Tumor-induced angiogenesis changes the angiogenic vessel density,$v$, through Eq. 9d:

$\frac{dv}{dt}=\beta_{ag}\left( \frac{c_{vegf}}{c_{vegf}+\alpha_{vegf0}} \right)-\omega_{ag}\left( 1-\frac{c_{vegf}}{c_{vegf}+\theta_{vegf0}} \right)v$

(9d)

where the first term is the production of angiogenic vessels in response to VEGF and the second term is vascular pruning in response to low VEGF conditions.

#### b) Tumor-induced suppressive factors (SFs):

$\frac{dc_{sf}}{dt}=\left( Transport_{matrix} c_{sf}+{J_{d}}_{sf,matrix} \right)+ \left( s_{sf}\left( C+\alpha_{T,C} \right) \right)-\left( U_{sf} \frac{c_{sf}}{c_{sf}+1} \right)c_{imAPC} (k_{1}\left[ for tissue \right], k_{2}\left[ for blood \right],k_{1}\gg k_{2})-\varepsilon_{sf} c_{sf}$

(10)

The first two terms are systemic transport of suppressive factors released by cancer. The Transport_matrix_ is the coefficient matrix of the set of equations1-8. ${J_{d}}_{sf,matrix}$ is the diagonal matrix of ${J_{d}}_{sf,j}$ in the set of equations1-7. The third term is the generation rate of these factors with cancer by a linear rate constant $s_{sf}$. The fourth term is uptake rate of SFs with immature APCs (imAPCs), and the last term is natural decay of SFs.$k_{1}$ is 1 and $k_{2}$ is 10^-3^.

#### c) Antigen and vaccination model:

$\frac{dc_{a}}{dt}=\left( Transport_{matrix} c_{a}+{J_{d}}_{a,matrix} \right)+s_{a_{tumor}}\left( C+\alpha_{T,C} \right)+s_{a_{vaccine}}-\left( U_{a} \frac{c_{a}}{c_{a}+1} \right)c_{imAPC} (k_{1}\left[ x Є tissue \right], k_{2}\left[ x Є blood \right],k_{1}\gg k_{2})-\varepsilon_{a} c_{a}$

(11)

$s_{a_{vaccine}}\left( x| R Є vaccination site, t=vaccination time (t_{v}) \right)=c_{0}' \delta(t-t_{v})$

(12)

In equation 11, the first two terms are systemic transport of antigen, the third term is production rate of antigen by cancer, the fourth term is uptake rate of antigen by immature APCs with maximum rate $U_{a}$ at tissue parts of all compartments. Antigen uptake by imAPCs was modeled with Michaelis–Menten kinetics to capture receptor-mediated saturation. The last term is clearance. In equation 12, vaccination is assumed as a Dirac delta function at vaccination time for each site of vaccination, including inner organs and skin regions.

#### d) Inflammatory Cytokines:

$\frac{dc_{ic}}{dt}=\left( Transport_{matrix}c_{ic}+{J_{d}}_{ic,matrix} \right)+ s_{ic} \left( c_{aAPC}+0.1 c_{imAPC}+0.1 \right)\left( 1+k_{a} \frac{c_{a}}{c_{a}+1} \right)\left[ x Є tissue \right]-\left( U_{ic} \frac{c_{ic}}{c_{ic}+1} \right)c_{imAPC} (k_{1}\left[ x Є tissue \right], k_{2}\left[ x Є blood \right],k_{1}\gg k_{2})-\varepsilon_{ic} c_{ic}$

(13)

The first two terms are systemic transport of inflammatory cytokines, the third term is production of cytokines by activated APC with maximum rate $s_{ic}$, by immature APCs with maximum rate 0.1 $s_{ic}$, and the rest of immune cells with maximum rate $s_{ic}$, the fourth term is uptake of cytokines with imAPCs, and the last term is decay.

#### e) T cell-induced activation Cytokines (IL-2):

$\frac{dc_{IL-2}}{dt}=\left( Transport_{matrix} c_{IL-2}+{J_{d}}_{IL-2,matrix} \right)+s_{IL-2} \left( \alpha_{1}c_{eT,CD4}+\alpha_{2} c_{eT,CD8} \right)-\left( U_{IL-2} \frac{c_{IL-2}}{c_{IL-2}+1} \right)c_{eT} \left( k_{1}\left[ x Є tissue \right], k_{2}\left[ x Є blood \right],k_{1}\gg k_{2} \right)-\varepsilon_{IL-2} c_{IL-2}$

(14)

The first two terms are systemic transport of activation cytokines, the third term is production of activation cytokines by effector T cells with a population distribution of $c_{eT,CD4}=\frac{2}{3}c_{eT} \& c_{eT,CD8}=\frac{1}{3}c_{eT}$ assumed that $\alpha_{1}\gg\alpha_{2}$ ($\alpha_{1}$ is 1 and $\alpha_{2}$ is 10^-3^), with a linear constant rate $s_{IL-2}$, the fourth term is uptake of cytokines with effector T cells, and the last term is decay.

#### f) Immature APCs (imAPC) and activated APCs (aAPCs):

$\frac{dc_{imAPC}}{dt}=Transport_{matrix} c_{imAPC}+ s_{imAPC} \left( 1+\beta_{1APC}\frac{c_{ic}}{c_{ic}+1} \right) \left( k_{1}\left[ for Bone marrow \right], k_{2}\left[ for skin \right],k_{1}\gg k_{2} \right)-r_{APC} \left( 1+\beta_{2APC}\frac{c_{ic}}{c_{ic}+1} \right)\left( \frac{c_{a}}{c_{a}+1} \right) c_{imAPC} e^{-k_{sf} c_{sf}}\left( k_{1}\left[ for peripheral tissues \right], k_{2}\left[ for Spleen and LNs \right],k_{1}\gg k_{2} \right)-\varepsilon_{imAPC} c_{imAPC}$

(15)

The first term is systemic transport of imAPCs, the second term is production of imAPC by bone marrow and skin, the third term is conversion rate of imAPC to aAPC by antigen enhanced by inflammatory and hindered by SFs mostly in tissue parts of peripheral organs (tumor, lung, skin, gut, etc.), and the last term is decay.

$\frac{dc_{aAPC}}{dt}=Transport_{matrix} c_{aAPC}+r_{APC} \left( 1+\beta_{2APC}\frac{c_{ic}}{c_{ic}+1} \right)\left( \frac{c_{a}}{c_{a}+1} \right) c_{imAPC} e^{-k_{sf} c_{sf}}\left( k_{1}\left[ for peripheral tissues \right], k_{2}\left[ for Spleen and LNs \right],k_{1}\gg k_{2} \right)-\varepsilon_{aAPC} c_{aAPC}$

(16)

The first term is systemic transport of aAPCs, the second term is conversion rate of imAPC to aAPC, and the last term is decay.

#### g) Naïve T cells (nTs) and activated effector T cells (eTs):

$\frac{dc_{nT}}{dt}=Transport_{matrix} c_{nT}+ s_{thymus} \left( 1+\beta_{1nT}\frac{c_{ic}}{c_{ic}+1} \right)[ for left ventricle of heart] -r_{T} {\left( 1+\beta_{2nT} \frac{c_{a}}{c_{a}+1} \right) c}_{aAPC}c_{nT} H\left( c_{eT}^{th}-c_{eT} \right) e^{-k_{sf} c_{sf}}\left[ for lymphoid Organs (LNs and Spleen) \right]-\varepsilon_{nT} c_{nT}$

(17)

The first term is systemic transport of nTs, the second term is production of nTs by thymus enhanced by the inflammatory cytokines, the third term is conversion rate of nTs to eTs by aAPCs enhanced by antigen and hindered by SFs limited by eT concentration occurred in LNs and Spleen, and the last term is decay.

$\frac{dc_{eT}}{dt}=Transport_{matrix} c_{eT} +p_{eT}\left( \frac{c_{IL-2}}{c_{IL-2}+1} \right)c_{eT} H\left( c_{eT}^{th}-c_{eT} \right) \left[ for lymphoid Organs (LNs and Spleen) \right]+ r_{T} {\left( 1+\beta_{2nT} \frac{c_{a}}{c_{a}+1} \right) c}_{aAPC}c_{nT} H\left( c_{eT}^{th}-c_{eT} \right) e^{-k_{sf} c_{sf}}\left[ for lymphoid Organs (LNs and Spleen) \right]+ \left( k_{-1}+k_{2}\left( p_{r} \right) \right)\alpha_{T,C}-k_{1}\left( 1+\beta_{v}\frac{v}{v+\alpha_{v}} \right){c_{eT}}_{tumor}^{ins}C -\varepsilon_{eT} c_{eT}$

(18)

The first term is systemic transport of eTs, the second term is proliferation of eTs in LNs and Spleen by activation cytokines limited by the concentration of eTs, the third term is conversion rate of nTs to eTs by aAPCs enhanced by antigen and hindered by SFs limited by eT concentration occurred in LNs and Spleen, the fourth term is de-conjugation of eTs from cancer cells, which can occur immediately after generation of tumor/T cell complex ($\alpha_{T,C}$) at a constant rate; $k_{-1}$, or after killing cancer cells by eTs (with the reaction rate equals $k_{2}\left( p_{r} \right)$). The fifth term represents the binding of eTs to cancer cells and the last term is decay.

### **Transvascular and Lymphatic Transport of Agents**

**Molecular agents:**

For transvascular exchange of plasma and soluble molecular species, we use a modified pore model to consider the convectional and diffusional transmigration of particle across the angiogenic vessels ^2-7^:

$$L_{j}=S_{V_{j}} \left[ L_{p_{j}}(p_{lum_{j}}-p_{ins_{j}}-\sigma_{v}(\pi_{lum}-\pi_{ins})) \right]$$

$$J_{j}=L_{j} \left( 1-\sigma_{d,ij} \right)$$

${J_{d}}_{j}=\kappa_{j}S_{V_{j}} \left( {ci}_{j}^{b}-{ci}_{j}^{ins} \right)\frac{P_{j}}{e^{P_{j}}-1}$

(19)

In this model, $\sigma_{d,ij}$ defined in Eq. 20 as a function of particle size of *i^th^* solute, $d_{m_{i}}$, and effective pore size of vessel wall, $d_{p_{j}}$, is colloid osmotic (oncotic) reflection coefficient for solute into plasma and $\sigma_{v}$is average oncotic reflection coefficient of plasma proteins, $S_{V}$ surface area per unit volume for transvascular exchange, $p_{lum}$ is intravascular blood pressure, $p_{ins}$is interstitial fluid pressure (IFP), $\pi_{lum}$ and $\pi_{ins}$ are respectively oncotic pressures of the intravascular plasma and interstitial fluid, $\kappa_{i}$ is solute permeability coefficient of the vessel wall, $L_{p_{j}}$ is the hydraulic conductivity of the vessel wall. A uniform baseline solute permeability coefficient was assumed for soluble molecular agents, while molecule- and tissue-specific transport differences were captured through size-dependent reflection coefficients based on effective vascular pore sizes. In contrast, hydraulic conductivity was assigned in an organ-specific manner to account for differences in vascular architecture.

*P_j_* defined in Eq.21 is the transvascular Peclet number, the ratio of convection to diffusion across the vessel wall. In Eq.19,${ci}_{j}^{b}$ is the plasma concentration of species $i$ in the blood flow of organ $j$ and ${ci}_{j}^{ins}$ represents the concentration of species $i$ in the interstitial space of organ $j$.

$\sigma_{d,ij}=\left( 1-\left( 1-\frac{d_{m_{i}}}{d_{p_{j}}} \right)^{2} \right)^{2}$

(20)

$$P_{j}=(1-\sigma_{d,j})\left[ \frac{L_{p_{j}}}{\kappa_{j}}(p_{lum_{j}}-p_{ins_{j}}-\sigma_{v}(\pi_{lum}-\pi_{ins})) \right]$$

(21)

In table S, Interstitial and microvascular hydrostatic pressures were assigned representative organ-specific values compiled from the physiological literature and previous transport models and were selected to reproduce reported Starling pressure gradients across different tissues.

Organ-specific vessel wall hydraulic conductivities ($L_{p_{j}}$) were assigned representative physiological values in units of m·mmHg⁻¹·day⁻¹, with higher conductivities for fenestrated tissues (e.g., liver) and lower conductivities for restrictive barriers (e.g., brain).

In table S, Effective vascular pore sizes were assigned to represent the permeability characteristics of each organ's microvasculature rather than the anatomical dimensions of endothelial openings.

**Cellular agents:**

We assume that the captured cells are in a quasi-steady state, such that their rate of accumulation on the vascular endothelium is negligible $\left( \frac{d{ci}_{j}^{c}}{dt} \approx0 \right)$. Under this assumption, the captured-cell equation can be eliminated, and an effective transmigration rate is derived as $J_{eff}=J\frac{a_{j}}{d_{j}+J}$. Because cellular transmigration, $J$, is an active biological process and is not governed solely by passive mechanical transport forces, we use the physiological transmigration constant reported in our previous study^1^. Detachment of captured cells from the vascular wall was neglected ($d_{j}\approx0$), assuming that the captured state represents firm adhesion preceding transendothelial migration. Thus, captured cells either remain transiently attached or proceed to transmigration, and detachment back into the bloodstream is not explicitly represented.

### **Model validation**

We use multi-step validation including A) interactions of T cells with APCs under the effect of antigen level, B) effect of tumor-induced suppressive factors (SFs) on APC activation and activation of T cells with APCs, and C) validation of post-vaccination activation in humans.

A) We used published data on T cell behavior under varying antigen concentrations in the presence of antigen-presenting cells (APCs). Specifically, we parameterized the conversion rates from naive T cells (nT) to effector T cells (eT) based on experimental observations reported by Bousso and Robey ^8^. In our model, we considered both freely migrating T cells (nT) and T cells in contact with APCs. Upon contact, T cells could either become activated or return to the naive state. The conversion rates from nT to eT were calibrated using these data. We then compared the model outputs for CD8⁺ T cell activation under conditions of no antigen, low antigen, and high antigen presentation (Figure S1.A). The model predictions showed good agreement with the corresponding experimental results.

**Figure S1. Model validation (calibration).** A) interactions of T cells with APC under the effect of antigen level ^8^, B) effect of tumor-induced suppressive factors (SFs) on APC activation and activation of T cells with APC^9^, and C) validation of post-vaccination activation in human^10^.

B) We validated the model’s suppressive SF terms using published data showing that tumor-derived IL-10 induces miR-187 and suppresses NF-κB–dependent cytokines (notably TNF-α, IL-6 and IL-12p40) ^9^. In primary human monocytes, IL-10 drives miR-187 expression and miR-187 reduces LPS-induced TNF-α both by destabilizing TNF-α mRNA and by down-modulating IκBζ ^9^. Independent studies demonstrate that IL-10 also prevents DC maturation (reduced MHC-II, CD80/CD86) and impairs DC-mediated priming of CD4⁺/CD8⁺ T cells^11,12^, supporting our model structure in which SFs such as IL-10 and miR-187 curb APC activation and consequently T-cell activation. The model predictions showed good agreement with the corresponding experimental results (Figure S1.B). For this validation, we correlated activation potential (AP) in our model used in Eqs. 15-18 as an exponential term with normalized values of TNF-α in the experimental study of Rossato, et al. ^9^.

C) We used a clinical study of T cell activation pre- and post-vaccination^10^ to validate the long-term response of vaccination in our model, comparing it to melanoma patients (Fig. S1C). Because the study did not report antigen or SF levels in patients, we applied a Bayesian machine learning optimization algorithm to simulate different antigen and SF levels, calibrating the model to match pre-vaccination T cell levels in tumors (TIH in our model). This calibrated pre-vaccine model then served as a control for comparison with post-vaccination data. In the referenced study^10^, patients received repeated intranodal injections of personalized RNA neoantigen vaccines, with tumor tissue collected before and after vaccination to analyze immune infiltration, corresponding to TIH in our model. Consistent with these observations, our model reproduces the post-vaccination increase in CD8⁺ T cell infiltration (Fig. S1C). Error bars in the simulation represent fluctuations due to varying tumor-induced antigen and SF levels, which show reduced sensitivity post-vaccination compared to pre-vaccination.

We further validated the model using two groups of patients vaccinated with peptide formulations either containing or lacking CpG adjuvant. CpG is known to enhance antigen-presenting cell (APC) activation through TLR9 signaling, promoting APC maturation and antigen presentation, and consequently enhancing Effector T-cell (eT) priming. Therefore, for the patient group receiving CpG adjuvant (Group A), the APC activation rate, $r_{APC}$, was assumed to be high (100-fold the baseline value listed in Table S), whereas for the group without CpG adjuvant (Group B), $r_{APC}$was assumed to be very low (0.01-fold the baseline value in Table S).

The clinical validation dataset consisted of ${HLA-A2}^{+}$ melanoma patients receiving four monthly subcutaneous vaccinations of Melan-A peptide formulated with IFA, either with or without CpG 7909 adjuvant. Peripheral blood samples were collected before vaccination and 7–10 days after the second and fourth vaccination cycles for quantification of antigen-specific eT cells. Accordingly, in the simulations, we considered melanoma located in the left upper body compartment and vaccination administered subcutaneously in the contralateral lower body compartment to mimic distal vaccination and lymphatic drainage. Vaccination timing in the simulations followed the clinical protocol with repeated monthly administrations initiated after establishment of the tumor. Effector T-cell (eT) levels in the bloodstream were then quantified 7–10 days after the second and fourth vaccination cycles for comparison with the experimental measurements.

To account for inter-patient variability, the T-cell activation rate for each patient was allowed to vary within approximately one order of magnitude around the baseline value to reproduce the experimentally observed pre-vaccination activation levels. In addition, systemic antigen and suppressive factor (SF) levels were adjusted for each patient such that the simulated baseline activation remained close to the corresponding experimental measurements before vaccination.

As shown in Fig. S2, the model predictions were in good agreement with the experimental measurements for both patient groups following two and four vaccination cycles, with average RMSE_log10 values of 0.1 and 0.048, and average MAE_1og10 values of 0.09 and 0.046, respectively. Because the clinical dataset does not provide sufficient patient-specific biological information, the model was not fully individualized. A fully patient-specific framework would require additional experimental and clinical parameters, including APC activation levels, systemic antigen burden, suppressive factor concentrations, and T-cell activation kinetics.

For this validation, we therefore assumed that the APC activation rate was fixed within each CpG treatment group. The clinical study did not provide sufficient patient-specific information for other sensitive model parameters identified in the sensitivity analysis, including systemic antigen levels and suppressive factor (SF) levels. Therefore, these parameters were phenomenologically calibrated within biologically reasonable ranges to reproduce the experimentally observed baseline responses prior to vaccination (“Before” in the plots). Subsequent simulations of the responses after the second and fourth vaccination cycles were then compared against the reported clinical measurements. This validation suggests that, if key patient-specific parameters—particularly systemic antigen and suppressive factor levels prior to vaccination—can be experimentally measured, the model may be used to predict individual patient responses to vaccination.

**Figure S2. Validation of the model against clinical vaccine-induced** $\mathbf{CD8}^{+}$ **T-cell activation data.**
(A) Comparison between experimental and simulated percentages of effector T cells (eT) in the bloodstream for ${HLA-A2}^{+}$ melanoma patients receiving Melan-A peptide vaccination formulated with IFA and CpG 7909 adjuvant. eT levels are shown before vaccination and 7–10 days after the second and fourth vaccination cycles. The accompanying table summarizes the root mean square error (RMSE) and mean absolute error (MAE) values calculated in $\log_{10}$ scale between experimental and simulated results for each patient and the overall average. (B) Comparison between experimental and simulated percentages of effector T cells (eT) in the bloodstream for ${HLA-A2}^{+}$ melanoma patients receiving Melan-A peptide vaccination formulated with IFA without CpG 7909 adjuvant. eT levels are shown before vaccination and 7–10 days after the second and fourth vaccination cycles. The accompanying table summarizes the RMSE and MAE values in $\log_{10}$ scale between experimental and simulated results for each patient and the overall average.

### **Effect of tumor-induced local pressure on vaccination on different parts of skin**

**Tumors can alter the distribution of soluble factors by increasing interstitial fluid pressure and flow in superficial network.** Tumor vessel leakiness can increase local tissue fluid pressure, which can alter fluid flow through the tissue and into the lymphatic system. To investigate this, we modeled the potential impact of this process on vaccination outcomes in cases where a superficial tumor elevates local fluid pressure. In our simulations, we increased the pressure of the RUB and infused vaccines separately into the RUB and the LUB. To isolate the effect of the injection site, we assumed that the tumor had no antigen source. Our results show that vaccinating the superficial skin close to the tumor (high-pressure region) compared to the opposite side reduces antigen accumulation in local LNs and lowers T cell activation in those LNs. This leads to a reduction in the total number of activated T cells in the tumor defined as tumor hotness (TH). These findings suggest that, in cases where a tumor increases local superficial pressure, vaccination on the side opposite the tumor may be more effective. When the vaccine is injected into high-pressure skin, it is more rapidly drained toward other skin sites, reducing the chance for the local superficial LNs to be activated—both for APC activation and for T cell activation. This drainage delay results in fewer T cells being presented to the TME within the same time frame. In contrast, our results for normal skin with constant and uniform pressure across regions indicate that there is no inherent priority for one skin site over another as the vaccination site.

**Figure S3. Impact of vaccination site on skin with pressure variations induced by a superficial tumor.** Red curves represent vaccination in tumor-affected high-pressure skin (HP), while blue curves correspond to vaccination in normal-pressure skin (NP).

### **Sensitivity Analysis to Identify Influential Parameters**

We performed a local sensitivity analysis to evaluate the effects of key model parameters on tumor hotness (TIH), including the release rate of suppressive factors (SF) by the tumor, $S_{SF}$; the release rate of tumor antigen, $S_{a}$; the production rate of inflammatory cytokines by immune cells, $S_{ic}$; the production rate of IL-2 by activated T cells, $S_{IL2}$; the production rate of antigen-presenting cells (APCs) by bone marrow and skin, $S_{imAPC}$; the production rate of naïve T cells by the thymus, $S_{thyme}$; the conversion rate of immature APCs to activated APCs, $r_{APC}$; the conversion rate of naïve T cells to effector T cells, $r_{T}$; and the IL-2–mediated proliferation rate of effector T cells, $P_{eT}$. The results are shown in Fig. S4. Each parameter was independently perturbed by ±20% relative to its baseline value listed in Table S. For the baseline simulation, we assumed a patient with intermediate levels of tumor-derived suppressive factor and antigen release rates ($S_{SF}=S_{a}={10}^{-3} \min^{-1}$), without vaccination, in order to avoid cross-effects between endogenous tumor antigen and vaccine-derived antigen.

The sensitivity analysis demonstrated that TIH was most sensitive to the tumor-derived suppressive factor release rate ($S_{SF}$) and tumor antigen release rate ($S_{a}$). Increasing $S_{SF}$by 20% reduced TIH by approximately 11%, whereas decreasing $S_{SF}$increased TIH by approximately 14%, highlighting the dominant inhibitory role of tumor-mediated immunosuppression. In contrast, increasing $S_{a}$increased TIH by approximately 10%, while decreasing $S_{a}$reduced TIH by approximately 13%, demonstrating the critical role of antigen availability in sustaining anti-tumor immune activation.

Strong sensitivity was also observed for parameters associated with T-cell generation and activation, including the naïve-to-effector T-cell conversion rate ($r_{T}$) and thymic naïve T-cell production rate ($S_{thyme}$), both of which altered TIH by approximately 7–8%. Moderate sensitivity was observed for APC-related processes, including the immature-to-activated APC conversion rate ($r_{APC}$) and immature APC production rate ($S_{imAPC}$). In comparison, inflammatory cytokine production ($S_{ic}$), IL-2 production ($S_{IL2}$), and IL-2–mediated effector T-cell proliferation ($P_{eT}$) produced relatively smaller changes in TIH, indicating lower local sensitivity around the selected baseline parameter set.

Overall, these results suggest that tumor hotness in the model is primarily governed by the balance between tumor-induced immunosuppression and systemic immune activation, while cytokine-mediated amplification mechanisms play comparatively secondary roles within the investigated parameter range. The substantial changes in TIH resulting from perturbations of $S_{SF}$, $S_{a}$, $r_{T}$, and $S_{thyme}$further indicate that these parameters are identifiable within the current modeling framework, as variations in their values produce distinguishable and non-negligible effects on model predictions.

**Fig. S4. Local sensitivity analysis of model parameters on tumor Immune hotness (TIH).** Each parameter was independently perturbed by ±20% relative to its baseline value listed in Table S, and the resulting percentage change in TIHwas quantified. The analyzed parameters include the tumor suppressive factor release rate ($S_{SF}$), tumor antigen release rate ($S_{a}$), inflammatory cytokine production rate by immune cells ($S_{ic}$), IL-2 production rate by activated T cells ($S_{IL2}$), immature APC production rate by bone marrow and skin ($S_{imAPC}$), naïve T-cell production rate by the thymus ($S_{thyme}$), immature-to-activated APC conversion rate ($r_{APC}$), naïve-to-effector T-cell conversion rate ($r_{T}$), and IL-2–mediated effector T-cell proliferation rate ($P_{eT}$). Positive values indicate an increase in TIH, whereas negative values indicate a reduction in TIH relative to the baseline simulation. The results demonstrate that TIH is most sensitive to tumor-derived suppressive factor release and tumor antigen release, followed by T-cell activation and thymic T-cell production, highlighting the dominant roles of tumor-mediated immunosuppression and systemic immune activation in regulating tumor immune hotness.

# **Data Availability Statement**

The data and MATLAB code supporting the findings of this study are available within the article and its Supporting Information.

### **Table S. Model parameters**

| cell adhesion | Description | constant | Ref |
| --- | --- | --- | --- |
| $a_{tumor}$ | Constant attachment rate constant of T cell to the blood vessel wall of tumor ;  Variable attachment rate of tumor: *a’_tumor =_ a_tumor_(V/(1+V)*) *, V : tumor volume* | 6.9×10^-3^ min^-1^  0 for nT | ^13^ |
| $a_{lung}$ | Attachment rate of T cell/APC to the blood vessel wall in lung | 3.1×10^-6^ min^-1^  0 for nT | ^13^ |
| $a_{liver}$ | Attachment rate of T cell/APC to the blood vessel wall in liver | 7.6×10^-9^ min^-1^  0 for nT | ^13^ |
| $a_{spleen}$ | Attachment rate of T cell/APC to the blood vessel wall in spleen | 8.1×10^-6^ min^-1^ | ^13^ |
| $a_{j}$ | Attachment rate of T cell/APC to the blood vessel wall in other tissues (*j^th^* organ) | 1×10^-6^ min^-1^  0 for nT | estimated |
| $a_{LNs}$ | Attachment rate of T cell/APC to the blood vessel wall in LNs | 8.1×10^-6^ min^-1^,  0 for activated T cells/APCs | estimated |
| $d_{j}$ | detachment rate of T cell/APC from the blood vessel wall of all tissues | $\approx$0 min^-1^ | estimated |
| natural decay | Description | constant | Ref |
| $\varepsilon_{a}$ | Antigen natural decay rate in tissues and LNs interstitium | Liver = 0.06 day^-1^  kidney = 0.04 day^-1^  Other organs = 0.033 day^-1^ | ^16,17^ |
| $\varepsilon_{SF}, \varepsilon_{ic} ,\varepsilon_{IL-2}$ | cytokines natural decay rate in tissues and LNs interstitium | 8.3 day^-1^ | ^18,19^ |
| $\varepsilon_{eT}$ | eT cells natural decay rate | 0.1 day^-1^ | ^20,21^ |
| $\varepsilon_{nT}$ | nT cells natural decay rate | 0.01 day^-1^ | ^22^ |
| $\varepsilon_{aAPC}$ | aAPCs natural decay rate | 0.231 day^-1^ | ^23-25^ |
| $\varepsilon_{imAPC}$ | imAPCs natural decay rate | 0.0495 day^-1^ | ^25-27^ |
| Blood flow rate | Description | constant | Ref |
| $Q_{tumor}$ | Initial Blood flow rate of tumor | 0.564 ml.min^-1^ | ^13^ |
| $Q_{skin}$ | Blood flow rate of skin | 220 ml.min^-1^ | ^13^ |
| $Q_{muscle}$ | Blood flow rate of muscle | 413 ml.min^-1^ | ^13^ |
| $Q_{bone}$ | Blood flow rate of bone | 138 ml.min^-1^ | ^28^ |
| $Q_{liver}$ | Blood flow rate of liver (hepatic portal vein from G.I. and spleen, and hepatic artery) | 800 ml.min^-1^ | ^28^ |
| $Q_{spleen}$ | Blood flow rate of spleen | 138 ml.min^-1^ | ^28^ |
| $Q_{int estine}$ | Blood flow rate of intestine | 468 ml.min^-1^ | ^28^ |
| $Q_{kidney}$ | Blood flow rate of kidney | 630 ml.min^-1^ | ^28^ |
| $Q_{brain}$ | Blood flow rate of brain | 300 ml.min^-1^ | ^1^ - ^28^ |
| $Q_{cardicvessels}$ | Blood flow rate of cardiac vessels | 120 ml.min^-1^ | ^1^ - ^28^ |
| Blood and lymphatic flow fraction | Description | constant | Ref |
| $f_{j}$ | Fractional flow rate of blood into vessels of tissue part | 0.99 | ^1^ |
| $ff_{j}$ | Fractional rate of lymphatic flow from tissue part to the main lymphatic flow | 0.99 | ^1^ |
| $f{f^{'}}_{j}$ | Fractional rate of lymphatic flow from tissue part of ULNs to the main lymphatic flow | 0.9 | ^1^ |
| Agents ($ci$ = *i^th^* cell) fraction from interstitium to lymphatic flow | Description | constant | Ref |
| $f_{ci,j}$ | T cell and APC fraction from tissue interstitium of all compartments except tumor to lymphatic flow | 1 | ^29^ |
| $f_{ci,tumor}$ | T cell fraction from tissue interstitium of tumor to lymphatic flow | 0 | ^29^ |
| $f_{ci,LNj}$ | T cell and APC fraction from LNs interstitium to lymphatic flow | 0.018 | ^29^ |
| Agents ($ci$ = *i^th^* molecule) fraction from interstitium to lymphatic flow | Description | constant | Ref |
| $f_{ci,j}$ | Molecular fraction from tissue interstitium of all compartments to lymphatic flow | 1 | ^1^-^29^ |
| $f_{ci,LNj}$ | Molecular fraction from LNs interstitium to lymphatic flow | 0.18 | ^1^-^29^ |
| Volume of tissue interstitium | Description | constant | Ref |
| $\boldsymbol{V}_{\boldsymbol{tumor}}^{\boldsymbol{ins}}$ | Averaged interstitium volume of tumor | 10.9 ml | ^13^ |
| ${\boldsymbol{V}^{\boldsymbol{ins}}}_{\boldsymbol{liver}}$ | Averaged interstitium volume of liver | 361.8 ml | ^28^ |
| ${\boldsymbol{V}^{\boldsymbol{ins}}}_{\boldsymbol{spleen}}$ | Averaged interstitium volume of spleen | 34.7 ml | ^28^ |
| ${\boldsymbol{V}^{\boldsymbol{ins}}}_{\boldsymbol{intestine}}$ | Averaged interstitium volume of intestine | 373.2 ml | ^28^ |
| ${\boldsymbol{V}^{\boldsymbol{ins}}}_{\boldsymbol{kidney}}$ | Averaged interstitium volume of kidney | 96.6 ml | ^28^ |
| ${\boldsymbol{V}^{\boldsymbol{ins}}}_{\boldsymbol{brain}}$ | Averaged interstitium volume of brain | 279 ml | ^1^ |
| ${\boldsymbol{V}^{\boldsymbol{ins}}}_{\boldsymbol{cardicvessels}}$ | Averaged interstitium volume of cardiac vessels | 42.9 ml | ^1^ |
| ${\boldsymbol{V}^{\boldsymbol{ins}}}_{\boldsymbol{lung}}$ | Averaged interstitium volume of normal part of lung | 299.7 ml | ^28^ |
| ${\boldsymbol{V}^{\boldsymbol{ins}}}_{\boldsymbol{bone}}$ | Averaged interstitium volume of bone | 279 ml | ^28^ |
| ${\boldsymbol{V}^{\boldsymbol{ins}}}_{\boldsymbol{muscle}}$ | Averaged interstitium volume of muscle | 4558 ml | ^28^ |
| ${\boldsymbol{V}^{\boldsymbol{ins}}}_{\boldsymbol{skin}}$ | Averaged interstitium volume of skin | 227 ml | ^28^ |
| Volume of LN interstitium | Description | constant | Ref |
| $\boldsymbol{V}_{\boldsymbol{LNs}}^{\boldsymbol{ins}}$ | Averaged interstitium volume of LNs | 34.7 ml | ^13^ |
| Volume of vascular tissue | Description | constant | Ref |
| $\boldsymbol{V}_{\boldsymbol{tumor}}^{\boldsymbol{b}}$ | Averaged vascular volume of tumor | 1.4 ml | ^13^ |
| ${\boldsymbol{V}^{\boldsymbol{b}}}_{\boldsymbol{liver}}$ | Averaged vascular volume of liver | 180.9 ml | ^28^ |
| ${\boldsymbol{V}^{\boldsymbol{b}}}_{\boldsymbol{spleen}}$ | Averaged vascular volume of spleen | 17 ml | ^28^ |
| ${\boldsymbol{V}^{\boldsymbol{b}}}_{\boldsymbol{intestine}}$ | Averaged vascular volume of intestine | 43 ml | ^28^ |
| ${\boldsymbol{V}^{\boldsymbol{b}}}_{\boldsymbol{kidney}}$ | Averaged vascular volume of kidney | 28.4 ml | ^28^ |
| ${\boldsymbol{V}^{\boldsymbol{b}}}_{\boldsymbol{brain}}$ | Averaged vascular volume of brain | 150 ml | ^1^ |
| ${\boldsymbol{V}^{\boldsymbol{a}}}_{\boldsymbol{Heart}}$ | Averaged volume of arterial cardiac vessels | 69.9 ml | ^1^ |
| ${\boldsymbol{V}^{\boldsymbol{v}}}_{\boldsymbol{Heart}}$ | Averaged volume of venus cardiac vessels | 60.6 ml | ^1^ |
| ${\boldsymbol{V}^{\boldsymbol{b}}}_{\boldsymbol{lung}}$ | Averaged vascular volume of normal part of lung | 99.9 ml | ^28^ |
| ${\boldsymbol{V}^{\boldsymbol{b}}}_{\boldsymbol{bone}}$ | Averaged vascular volume of bone | 150 ml | ^28^ |
| ${\boldsymbol{V}^{\boldsymbol{b}}}_{\boldsymbol{muscle}}$ | Averaged vascular volume of muscle | 700 ml | ^28^ |
| ${\boldsymbol{V}^{\boldsymbol{b}}}_{\boldsymbol{skin}}$ | Averaged vascular volume of skin | 462 ml | ^28^ |
| Volume of vascular LN | Description | constant | Ref |
| $\boldsymbol{V}_{\boldsymbol{LNs}}^{\boldsymbol{b}}$ | Averaged vascular volume of LNs | 17 ml | ^13^ |
| Immune cell reactions | Description | constant | Ref |
| $s_{Thymus}$ | nT cell production rate by thymus | 3.33×10^2^ day^-1^ mm^-3^ | ^30,31^ |
| $s_{imAPC}$ | aAPC cell production rate by bone marrow | 10^3^ day^-1^ mm^-3^ | ^32,33^ |
| $\beta_{1nT}$ | Enhancement factor for thymus production of nTs with inflammatory cytokines | 8 | ^34-36^ |
| $\beta_{2nT}$ | Enhancement factor for activation of T cells with antigen | 10 | ^37,38^ |
| $\beta_{1APC}$ | Enhancement factor for bone marrow production of imAPCs with inflammatory cytokines | 5 | ^39,40^ |
| $\beta_{2APC}$ | Enhancement factor for activation of APCs with antigen | 1 | ^39,40^ |
| $p_{eT}$ | eT cell proliferation rate in LNs and the Spleen | 0.01 day^-1^ | ^41-43^ |
| $r_{T}$ | baseline rate of nT to eT conversion | 0.1 day^-1^ | ^20,44,45^ |
| $r_{APC}$ | baseline maturation/activation rate of imAPC to aAPC | 14.4 day^-1^ | ^40,46,47^ |
| $c_{eT}^{th}$ | eT cell capacity of LNs | 10^3^ ml | ^1^ |
| $s_{a}$ | Production rate of antigen by cancer cells | 10^-3^ min^-1^ | ^1,48^ |
| $s_{sf}$ | Production rate of SF by cancer cells | 10^-3^ min^-1^ | ^49^ |
| $s_{ic}$ | Production rate of inflammatory cytokines by immune cells | 10^-5^ min^-1^ | ^50^ |
| $k_{a}$ | Enhancement factor of cytokine production with antigen | 20 | Estimated |
| $s_{IL-2}$ | Production rate of activation cytokines by activated T cells | 1.5 day^-1^(cell/ml)^-1^ | ^51,52^ |
| $\alpha_{1}, \alpha_{2}$ | Ratios of IL-2 production with CD4 (α_1_) vs CD8 eTs (α_2_) | α_1 = 1,_ α_2 =_ 10^-3^ | ^51,52^ |
| $U_{a}$ | Uptake rate of antigen by imAPC | 10^-3^ day^-1^ | ^53^ |
| $U_{ic}$ | Uptake rate of inflammatory cytokines by imAPC | 10^-5^ day^-1^ | ^50,53^ |
| $U_{IL-2}$ | Uptake rate of activation cytokines by eT | 10^-1^ day^-1^ | ^50,53,54^ |
| Tumor-immune interaction | Description | constant | Ref |
| $k_{1}$ | T cell /tumor cell complex formation rate | 1.3 ×10^-7^ day^-1^ (cell/ml)^-1^ | ^55^ |
| $k_{-1}$ | T cell /tumor cell complex degeneration rate | 24 day^-1^ | ^55^ |
| $k_{2}$ | T cell/tumor cell complex stabilization rate | 7.2 day^-1^ | ^55^ |
| $p_{r}$ | Probability of T cell-induced cancer killing | 0 | ^1^- ^55^ |
| $\beta_{v}$ | maximum value to enhance the accessibility of T cells to tumor cells by angiogenic vessels | 10 | ^1^- ^55^ |
| $\alpha_{v}$ | M-M constant to enhance the accessibility of T cells to tumor cells by angiogenic vessels | 1 | ^55^ |
| Tumor growth and Angiogenesis | Description | constant | Ref |
| $g_{v}$ | Maximum rate of vascular tumor growth | 1.4×10^-3^ min^-1^ | ^1^ -^56,57^ |
| $g_{av}$ | Maximum rate of avascular tumor growth | 7×10^-7^ min^-1^ | ^1^ -^56,57^ |
| $\lambda_{a0}$ | M-M constant for Maximum avascular tumor growth | 1 | ^1^ -^56,57^ |
| $\eta_{ag}$ | Production rate of tumor-induced VEGF | 1.1×10^-10^ min^-1^ | ^1^-^58,59^ |
| $\varepsilon_{vegf}$ | Natural decay rate of tumor-induced VEGF | 7×10^-5^ min^-1^ | ^1^-^58,59^ |
| $\beta_{ag}$ | Maximum rate of angiogenesis | 1.4×10^-4^ min^-1^ | ^1^-^58,59^ |
| $\alpha_{vegf0}$ | M-M constant of angiogenesis rate | 1×10^-6^ | ^1^-^58,59^ |
| $\omega_{ag}$ | Maximum value of angiogenic vessel degradation | 3.3×10^-3^ min^-1^ | ^1^-^58,59^ |
| $\theta_{vegf0}$ | M-M constant of angiogenic vessel degradation | 3.3×10^-7^ | ^1^-^58,59^ |
| Hemodynamics and interstitial fluid Description constant Ref | | | |
| $L_{p_{j}}$ | hydraulic conductivity of vessels wall | Lung = 8.64×10^-5^ m/(mmHg.day)  Liver = 4×10^-4^ m/(mmHg.day)  Kidney = 1×10^-4^ m/(mmHg.day)  Brain (BBB) = 8.64×10^-6^ m/(mmHg.day)  Skins (1-6) = 1×10^-5^ m/(mmHg.day)  Muscle = 1×10^-5^ m/(mmHg.day)  Bone = 5×10^-6^ m/(mmHg.day)  Intestine (G.I.) = 2×10^-5^ m/(mmHg.day)  Spleen = 5×10^-5^ m/(mmHg.day)  Tumor = 9×10^-5^ m/(mmHg.day) | ^3,60,61^ |
| $p_{lum_{j}}$ | Intravascular pressure | Lung = 15 mmHg  Liver = 8 mmHg  Kidney = 40 mmHg  Brain = 20 mmHg  Skins (1-6) = 25 mmHg  Muscle = 25 mmHg  Bone = 13 mmHg  Intestine (G.I.) = 15 mmHg  Spleen = 12 mmHg  Tumor = 35 mmHg | ^62,63^ |
| $p_{ins_{j}}$ | Interstitial pressure | Lung = 0 mmHg  Liver = 5 mmHg  Kidney = 10 mmHg  Brain = 10 mmHg  Skins (1-6) = 1 mmHg  Muscle = 1 mmHg  Bone = 1 mmHg  Intestine (G.I.) = 3 mmHg  Spleen = 2 mmHg  Tumor = 25 mmHg | ^62,64-66^ |
| $\sigma_{v}$ | average oncotic reflection coefficient of plasma proteins | 0.91 for healthy tissue and 0.82 for tumor tissue | ^67^, ^68^ |
| $\pi_{lum}$ | collide osmotic (oncotic) pressures of intravascular plasma | 20 mmHg for both healthy and tumor tissues | ^67^, ^68^ |
| $\pi_{ins}$ | collide osmotic (oncotic) pressures of interstitial fluid | 10 mmHg for heathy tissue and 15 mmHg for tumor tissue | ^67^, ^68^ |
| $S_{V}$ | Characteristic value of surface area of neo-vessels per unit volume for mass transport in the interstitium | 7×10^3^m^-1^ for healthy tissue and 2×10^4^m^-1^ for tumor tissue | ^67^, ^68^ |
| $d_{p_{j}}$ | Effective vascular pore sizes | Tumor = 400 nm  Continuous capillaries (muscle, skin, brain) = 5 nm  Bone = 5 nm  Brain (BBB) = 1 nm  Lung microvasculature = 5 nm  Kidney = 15 nm  Liver = 100 nm  Spleen = 150 nm  Intestine = 8 nm | ^69-72^ |
| $d_{m_{j}}$ | Particle size of molecules (diameter) | 6 nm | ^73-76^ |
| $\kappa_{i}$ | solute permeability coefficient | 5.73×10^-9^ cm/s for tumor tissue  0.73×10^-9^ cm/s for normal tissues | ^4^ |

**References**

1	Nikmaneshi, M. R., Baish, J. W., Zhou, H., Padera, T. P. & Munn, L. L. Transport Barriers Influence the Activation of Anti‐Tumor Immunity: A Systems Biology Analysis. *Advanced Science* **10**, 2304076 (2023).

2	Nikmaneshi, M. R., Jain, R. K. & Munn, L. L. Computational simulations of tumor growth and treatment response: benefits of high-frequency, low-dose drug regimens and concurrent vascular normalization. *PLoS Computational Biology* **19**, e1011131 (2023).

3	Baxter, L. T. & Jain, R. K. Transport of fluid and macromolecules in tumors. I. Role of interstitial pressure and convection. *Microvascular research* **37**, 77–104 (1989).

4	Baxter, L. T. & Jain, R. K. Transport of fluid and macromolecules in tumors. II. Role of heterogeneous perfusion and lymphatics. *Microvascular research* **40**, 246–263 (1990).

5	Baxter, L. T. & Jain, R. K. Transport of fluid and macromolecules in tumors: III. Role of binding and metabolism. *Microvascular research* **41**, 5–23 (1991).

6	Baxter, L. T. & Jain, R. K. Transport of fluid and macromolecules in tumors. IV. A microscopic model of the perivascular distribution. *Microvascular research* **41**, 252–272 (1991).

7	Jain, R. K. Vascular and interstitial barriers to delivery of therapeutic agents in tumors. *Cancer and Metastasis Reviews* **9**, 253–266 (1990).

8	Bousso, P. & Robey, E. Dynamics of CD8+ T cell priming by dendritic cells in intact lymph nodes. *Nature immunology* **4**, 579–585 (2003).

9	Rossato, M. *et al.* IL-10–induced microRNA-187 negatively regulates TNF-α, IL-6, and IL-12p40 production in TLR4-stimulated monocytes. *Proceedings of the National Academy of Sciences* **109**, E3101–E3110 (2012).

10	Sahin, U. *et al.* Personalized RNA mutanome vaccines mobilize poly-specific therapeutic immunity against cancer. *Nature* **547**, 222–226 (2017).

11	Yang, A. S. & Lattime, E. C. Tumor-induced interleukin 10 suppresses the ability of splenic dendritic cells to stimulate CD4 and CD8 T-cell responses. *Cancer research* **63**, 2150–2157 (2003).

12	Commeren, D. L. *et al.* Paradoxical effects of interleukin‐10 on the maturation of murine myeloid dendritic cells. *Immunology* **110**, 188–196 (2003).

13	Zhu, H., Melder, R. J., Baxter, L. T. & Jain, R. K. Physiologically based kinetic model of effector cell biodistribution in mammals: implications for adoptive immunotherapy. *Cancer research* **56**, 3771–3781 (1996).

14	Melder, R. J., Salehi, H. A. & Jain, R. K. Interaction of activated natural killer cells with normal and tumor vessels in cranial windows in mice. *Microvascular research* **50**, 35–44 (1995).

15	Bjerknes, M., Cheng, H. & Ottaway, C. A. Dynamics of lymphocyte-endothelial interactions in vivo. *Science* **231**, 402–405 (1986).

16	Grommé, M. & Neefjes, J. Antigen degradation or presentation by MHC class I molecules via classical and non-classical pathways. *Molecular immunology* **39**, 181–202 (2002).

17	Roopenian, D. C. & Akilesh, S. FcRn: the neonatal Fc receptor comes of age. *Nature reviews immunology* **7**, 715–725 (2007).

18	Schmidt, F. M. *et al.* Inflammatory cytokines in general and central obesity and modulating effects of physical activity. *PloS one* **10**, e0121971 (2015).

19	Yiu, H. H., Graham, A. L. & Stengel, R. F. Dynamics of a cytokine storm. (2012).

20	Macallan, D. C. *et al.* Rapid turnover of effector–memory CD4+ T cells in healthy humans. *The Journal of experimental medicine* **200**, 255–260 (2004).

21	Macallan, D. C., Busch, R. & Asquith, B. Current estimates of T cell kinetics in humans. *Current Opinion in Systems Biology* **18**, 77–86 (2019).

22	This, S., Rogers, D., Gauthier, È. M., Mandl, J. N. & Melichar, H. J. in *Seminars in Immunology.* 101702 (Elsevier).

23	Kamath, A. T., Henri, S., Battye, F., Tough, D. F. & Shortman, K. Developmental kinetics and lifespan of dendritic cells in mouse lymphoid organs. *Blood, The Journal of the American Society of Hematology* **100**, 1734–1741 (2002).

24	Lubin, R. *et al.* The lifespan and kinetics of human dendritic cell subsets and their precursors in health and inflammation. *Journal of Experimental Medicine* **221**, e20220867 (2024).

25	Chen, M., Huang, L., Shabier, Z. & Wang, J. Regulation of the lifespan in dendritic cell subsets. *Molecular immunology* **44**, 2558–2565 (2007).

26	Merad, M. & Manz, M. G. Dendritic cell homeostasis. *Blood, The Journal of the American Society of Hematology* **113**, 3418–3427 (2009).

27	Cremer, I. *et al.* Long-lived immature dendritic cells mediated by TRANCE-RANK interaction. *Blood, The Journal of the American Society of Hematology* **100**, 3646–3655 (2002).

28	Zhu, H., Melder, R. J., Baxter, L. T. & Jain, R. K. Physiologically based kinetic model of effector cell biodistribution in mammals: implications for adoptive immunotherapy. *Cancer Res* **56**, 3771–3781 (1996).

29	Butcher, E. Following cellular traffic: methods of labelling lymphocytes and other cells to trace their migration in vivo. *Handbook of experimental immunology* **2**, 57.51 (1986).

30	Hazenberg, M. D., Borghans, J. A., de Boer, R. J. & Miedema, F. Thymic output: a bad TREC record. *Nature immunology* **4**, 97–99 (2003).

31	de Boer, R. J., Tesselaar, K. & Borghans, J. A. in *Seminars in Immunology.* 101839 (Elsevier).

32	Schirrmacher, V. Bone marrow: The central immune system. *Immuno* **3**, 289–329 (2023).

33	Rößner, S. *et al.* Myeloid dendritic cell precursors generated from bone marrow suppress T cell responses via cell contact and nitric oxide production in vitro. *European journal of immunology* **35**, 3533–3544 (2005).

34	Yan, F. *et al.* Thymic function in the regulation of T cells, and molecular mechanisms underlying the modulation of cytokines and stress signaling. *Molecular medicine reports* **16**, 7175–7184 (2017).

35	De Boer, R. J. & Perelson, A. S. Quantifying T lymphocyte turnover. *Journal of theoretical biology* **327**, 45–87 (2013).

36	Robert, P. A., Kunze-Schumacher, H., Greiff, V. & Krueger, A. Modeling the dynamics of T-cell development in the thymus. *Entropy* **23**, 437 (2021).

37	Wölfl, M. & Greenberg, P. D. Antigen-specific activation and cytokine-facilitated expansion of naive, human CD8+ T cells. *Nature protocols* **9**, 950–966 (2014).

38	Quenum, A. J. I., Santharam, M. A., Ramanathan, S. & Ilangumaran, S. Inflammatory Cytokines That Enhance Antigen Responsiveness of Naïve CD8+ T Lymphocytes Modulate Chromatin Accessibility of Genes Impacted by Antigen Stimulation. *International Journal of Molecular Sciences* **23**, 14122 (2022).

39	Bilate, A. M. & Lafaille, J. J. Induced CD4+ Foxp3+ regulatory T cells in immune tolerance. *Annual review of immunology* **30**, 733–758 (2012).

40	Shortman, K. & Naik, S. H. Steady-state and inflammatory dendritic-cell development. *Nature Reviews Immunology* **7**, 19–30 (2007).

41	Chin, S. *et al.* (2022).

42	Rollings, C. M., Sinclair, L. V., Brady, H. J., Cantrell, D. A. & Ross, S. H. Interleukin-2 shapes the cytotoxic T cell proteome and immune environment–sensing programs. *Science signaling* **11**, eaap8112 (2018).

43	Kamimura, D. & Bevan, M. J. Naive CD8+ T cells differentiate into protective memory-like cells after IL-2–anti–IL-2 complex treatment in vivo. *The Journal of experimental medicine* **204**, 1803–1812 (2007).

44	Zarnitsyna, V. I. *et al.* Dynamics and turnover of memory CD8 T cell responses following yellow fever vaccination. *PLoS Computational Biology* **17**, e1009468 (2021).

45	Reynolds, J., Coles, M., Lythe, G. & Molina-París, C. Mathematical model of naive T cell division and survival IL-7 thresholds. *Frontiers in Immunology* **4**, 434 (2013).

46	Banchereau, J. & Steinman, R. M. Dendritic cells and the control of immunity. *Nature* **392**, 245–252 (1998).

47	Poženel, P., Zajc, K. & Švajger, U. Factor of time in dendritic cell (DC) maturation: short-term activation of DCs significantly improves type 1 cytokine production and T cell responses. *Journal of translational medicine* **22**, 541 (2024).

48	Mohammad Mirzaei, N. *et al.* A PDE model of breast tumor progression in MMTV-PyMT mice. *Journal of Personalized Medicine* **12**, 807 (2022).

49	Wakefield, L. M. *et al.* Recombinant latent transforming growth factor beta 1 has a longer plasma half-life in rats than active transforming growth factor beta 1, and a different tissue distribution. *The Journal of clinical investigation* **86**, 1976–1984 (1990).

50	Murtaugh, M. P. & Foss, D. L. Inflammatory cytokines and antigen presenting cell activation. *Veterinary immunology and immunopathology* **87**, 109–121 (2002).

51	Paliard, X. *et al.* Simultaneous production of IL-2, IL-4, and IFN-gamma by activated human CD4+ and CD8+ T cell clones. *The Journal of Immunology* **141**, 849–855 (1988).

52	Zhang, J. *et al.* Inducible IL-2 production and IL-2+ cell expansion are landmark events for T-cell activation of teleost. *Fish & Shellfish Immunology* **148**, 109515 (2024).

53	Chou, H.-C., Chiu, S.-J. & Hu, T.-M. Quantitative analysis of macrophage uptake and retention of fluorescent organosilica nanoparticles: implications for nanoparticle delivery and therapeutics. *ACS Applied Nano Materials* **7**, 3656–3667 (2024).

54	Humblet-Baron, S. *et al.* IL-2 consumption by highly activated CD8 T cells induces regulatory T-cell dysfunction in patients with hemophagocytic lymphohistiocytosis. *Journal of Allergy and Clinical Immunology* **138**, 200–209. e208 (2016).

55	Matzavinos, A., Chaplain, M. A. & Kuznetsov, V. A. Mathematical modelling of the spatio‐temporal response of cytotoxic T‐lymphocytes to a solid tumour. *Mathematical Medicine and Biology* **21**, 1–34 (2004).

56	Watanabe, Y., Dahlman, E. L., Leder, K. Z. & Hui, S. K. A mathematical model of tumor growth and its response to single irradiation. *Theoretical Biology and Medical Modelling* **13**, 1–20 (2016).

57	Enderling, H. & AJ Chaplain, M. Mathematical modeling of tumor growth and treatment. *Current pharmaceutical design* **20**, 4934–4940 (2014).

58	Nikmaneshi, M. R., Firoozabadi, B., Mozafari, A. & Munn, L. L. A multi-scale model for determining the effects of pathophysiology and metabolic disorders on tumor growth. *Scientific reports* **10**, 1–20 (2020).

59	Nikmaneshi, M. R., Firoozabadi, B. & Mozafari, A. Chemo‐Mechanistic multi‐scale model of a three‐dimensional tumor microenvironment to quantify chemotherapy response of cancer. *Biotechnology and Bioengineering* (2021).

60	Michel, C. & Curry, F. Microvascular permeability. *Physiological reviews* (1999).

61	Jain, R. K. Transport of molecules across tumor vasculature. *Cancer and Metastasis Reviews* **6**, 559–593 (1987).

62	Scallan, J., Huxley, V. & Korthuis, R. Fluid movement across the endothelial barrier. *Morgan & Claypool Life Sciences* (2010).

63	Feher, J. J. *Quantitative human physiology: an introduction*. (Academic press, 2017).

64	Sven, K. & Josipa, F. Interstitial hydrostatic pressure: a manual for students. *Advances in physiology education* **31**, 116–117 (2007).

65	No, N. Y. N. Y. Y. Interstitial Hydrostatic Pressure: a Manual for Students During the learning of capillary fluid traffic, students are often puzzled about the negative values found in their text-books for interstitial pressures in subcutaneous tissue and lungs (4, 6). Positive pressures are reported for the brain, kidneys.

66	Hughes, D. Transvascular fluid dynamics. *Veterinary Anaesthesia and Analgesia* **27**, 63–69 (2000).

67	Soltani, M. & Chen, P. Numerical modeling of interstitial fluid flow coupled with blood flow through a remodeled solid tumor microvascular network. *PloS one* **8**, e67025 (2013).

68	Zhao, G. *et al.* Numerical simulation of blood flow and interstitial fluid pressure in solid tumor microcirculation based on tumor-induced angiogenesis. *Acta Mechanica Sinica* **23**, 477–483 (2007).

69	Sarin, H. Physiologic upper limits of pore size of different blood capillary types and another perspective on the dual pore theory of microvascular permeability. *Journal of angiogenesis research* **2**, 14 (2010).

70	Stylianopoulos, T. & Jain, R. K. Combining two strategies to improve perfusion and drug delivery in solid tumors. *Proceedings of the National Academy of Sciences* **110**, 18632–18637 (2013).

71	Dewhirst, M. W. & Secomb, T. W. Transport of drugs from blood vessels to tumour tissue. *Nature Reviews Cancer* **17**, 738–750 (2017).

72	Hobbs, S. K. *et al.* Regulation of transport pathways in tumor vessels: role of tumor type and microenvironment. *Proceedings of the National Academy of Sciences* **95**, 4607–4612 (1998).

73	Erickson, H. P. Size and shape of protein molecules at the nanometer level determined by sedimentation, gel filtration, and electron microscopy. *Biological procedures online* **11**, 32 (2009).

74	Ward, N. C. *et al.* IL-2/CD25: a long-acting fusion protein that promotes immune tolerance by selectively targeting the IL-2 receptor on regulatory T cells. *The Journal of Immunology* **201**, 2579–2592 (2018).

75	Hawe, A., Hulse, W. L., Jiskoot, W. & Forbes, R. T. Taylor dispersion analysis compared to dynamic light scattering for the size analysis of therapeutic peptides and proteins and their aggregates. *Pharmaceutical research* **28**, 2302–2310 (2011).

76	Hirvonen, L. M. *et al.* Hydrodynamic Radii of Ranibizumab, Aflibercept and Bevacizumab Measured by Time-Resolved Phosphorescence Anisotropy: Hirvonen et al. *Pharmaceutical Research* **33**, 2025–2032 (2016).
